# Urinary Volatile Compounds as Biomarkers for Lung Cancer: A Proof of Principle Study Using Odor Signatures in Mouse Models of Lung Cancer

**DOI:** 10.1371/journal.pone.0008819

**Published:** 2010-01-27

**Authors:** Koichi Matsumura, Maryanne Opiekun, Hiroaki Oka, Anil Vachani, Steven M. Albelda, Kunio Yamazaki, Gary K. Beauchamp

**Affiliations:** 1 Monell Chemical Senses Center, Philadelphia, Pennsylvania, United States of America; 2 Panasonic Corporation, Kyoto, Japan; 3 University of Pennsylvania Medical Center, Philadelphia, Pennsylvania, United States of America; East Carolina University, United States of America

## Abstract

A potential strategy for diagnosing lung cancer, the leading cause of cancer-related death, is to identify metabolic signatures (biomarkers) of the disease. Although data supports the hypothesis that volatile compounds can be detected in the breath of lung cancer patients by the sense of smell or through bioanalytical techniques, analysis of breath samples is cumbersome and technically challenging, thus limiting its applicability. The hypothesis explored here is that variations in small molecular weight volatile organic compounds (“odorants”) in urine could be used as biomarkers for lung cancer. To demonstrate the presence and chemical structures of volatile biomarkers, we studied mouse olfactory-guided behavior and metabolomics of volatile constituents of urine. Sensor mice could be trained to discriminate between odors of mice with and without experimental tumors demonstrating that volatile odorants are sufficient to identify tumor-bearing mice. Consistent with this result, chemical analyses of urinary volatiles demonstrated that the amounts of several compounds were dramatically different between tumor and control mice. Using principal component analysis and supervised machine-learning, we accurately discriminated between tumor and control groups, a result that was cross validated with novel test groups. Although there were shared differences between experimental and control animals in the two tumor models, we also found chemical differences between these models, demonstrating tumor-based specificity. The success of these studies provides a novel proof-of-principle demonstration of lung tumor diagnosis through urinary volatile odorants. This work should provide an impetus for similar searches for volatile diagnostic biomarkers in the urine of human lung cancer patients.

## Introduction

Lung cancer is the leading cause of cancer-related deaths throughout most of the world [1]. The only treatment that that achieves a high rate of cure is surgical resection of early disease (before metastatic spread occurs). Since only about 25% of cases are diagnosed at this early stage, effective early diagnostic techniques are urgently needed.

Aggressive and early chest imaging of high risk patients is emerging as the dominant approach to early diagnosis, although large studies to validate this approach are still ongoing [2], [3], [4]. Unfortunately, although imaging is quite sensitive, it is also relatively non-specific. Recent studies have shown that between 5–26% of high risk smoking patients have detectable lung nodules by CT screening, however only an average of about 4% (with a range of 2–11%) of these nodules are malignant [5]. Clearly surgical resection of all of these nodules is neither practical nor desirable. Approaches to determine which nodules should be removed are thus needed. One attractive strategy would be to combine a sensitive imaging technique with a biomarker of lung cancer to increase specificity [6], [7], [8]. Because the incidence of lung cancer in this “nodule population” is significantly higher than in current or former smoking populations, biomarkers in this context would not require the extremely high sensitivities and specificities needed for population screening. Another use for such a biomarker might be to follow the course of the tumor after treatment.

With the development of high-throughput techniques for biomarker discovery [9], the field of lung cancer biomarkers has recently expanded substantially. Current biomarker candidates from blood, sputum, and urine include many classes of molecules including proteins, tumor antigens, anti-tumor antibodies, cell type-specific peptides, various metabolic products, and epigenetic phenomena such as hyper-methylated DNA, RNA, and specific gene expression [10]. However, no biomarker identified to date has been shown to have adequate sensitivity, specificity and reproducibility to be considered sufficient for use to detect and monitor lung cancer development.

Another class of biomarkers for lung cancer could be small molecular weight volatile organic compounds. These molecules, which can be perceived as odors (especially by animals), have been shown to function as “signatures” that convey social, emotional and health information to other members of the species [11]. There might be two sources of volatile markers in lung cancer patients. Studies have shown that lung cancer cell lines can release specific volatile organic compounds *in vitro* [12]. The presence of a growing tumor could also induce specific metabolic or nutritional changes that could alter the production or release of such compounds [6].

The “volatile hypothesis” for lung cancer has led to a number of studies examining the utility of analyzing these compounds in exhaled breath using either animals (such as dogs) [13] or sophisticated biochemical techniques [14], [15]. Some of these studies have shown promise. For example, a recent study from the Chen group [16] using solid phase micro-extraction followed by gas chromatography showed that 1-butanol and 3-hydroxy-2-butanone were found at significantly higher concentrations in the breath of lung cancer patients compared to controls. Dragonieri et al. used an “electronic nose” and were able to discriminate patients with lung cancer versus those with chronic obstructive lung disease with relatively high sensitivity and specificity [17].

Unfortunately, collecting, handling, storing, concentrating and analyzing breath samples is cumbersome, technically challenging, and may thus not be easy to apply widely. A partial solution to these problems would be to use a much more convenient source of volatiles, such as urine samples although urine, like breath, will include not only endogenous volatiles but also exogenous ones from sources such as diet and the environment. In this regard, Willis et al. (2004) reported that dogs could be trained to distinguish patients with bladder cancer on the basis of urine odor more successfully than would expected by chance alone [18]. Unfortunately, a follow-up study by Gordon et1 al. [19] was unable to reproduce these findings in urine samples from patients with breast and prostate cancer.

Based on these considerations, the hypothesis explored in this paper is that variations in small molecular weight volatile organic compounds (“odorants”) in urine could be used as biomarkers for lung cancer. One of the primary difficulties in attempting to initially identify volatile biomarkers from human patients is the vast variation that can be due to uncontrolled variables such as genetic and dietary differences, personal care product usage, and other environmental variables that can impact on body odor volatiles. The observation that dogs can apparently filter out these potential distractions and focus on the disease signature (see above) suggests that potentially useful biomarkers may exist.

**Figure 1:**
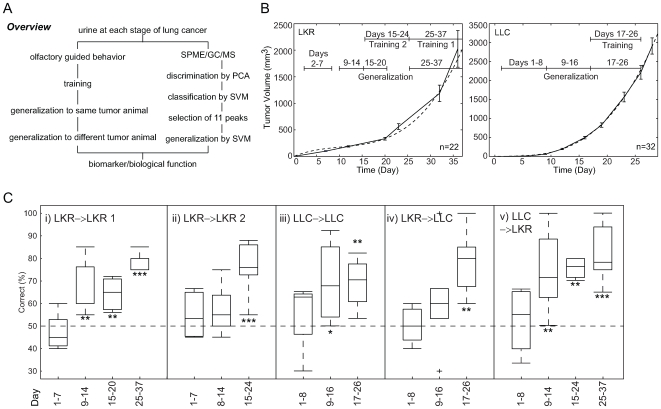
Tumor growth curves and urine collection times for bioassays and the results of bioassay. (A) Overview of experimental procedure. We employed mouse olfactory guided behavior (left) and metabolomic (right) approaches. (B) LKR cells and LLC cells were injected subcutaneously into the flanks of adult male mice and tumor size was measured weekly thereafter. Each time point shows the mean±SEM tumor size. Solid line: actual data; Dotted line: curve fitted with cubic function; LKR: y = 0.092*x^3^ − 2.8*x^2^+38*x − 18, LLC: y = 0.16*x^3^ − 0.83*x^2^+3.5*x − 4. Mouse urine was collected individually once a day and was used for chemical analysis and for bioassay during the periods indicated: For LKR - Days 15−24 and 25−37 for training and Days 2−7, 9−14, 15−20, and 25−37 for generalization; For LLC - Days 17−26 for training and Days 1−8, 9−16, and 17−26 for generalization. (C) Box plot of generalization scores for bioassay and the correlations among tests. Blue boxes represent the lower and upper quartiles. The red horizontal bar in each box indicates the median. The dotted line represents the range of observations. The plus (+) marks extreme outlier observations. *;*P*<0.01, **;*P*<0.001, ***;*P*<0.0001 compared to the null hypothesis of a 50% generalization score. From left, LKR-trained mouse urine generalization to LKR mouse urine (Training 1, Figure 1C-i); LKR- trained mouse urine generalization to LKR mouse urine (Training 2, Figure 1C-ii); LLC-trained mouse urine generalization to LLC mouse urine (Figure 1C-iii); LKR-trained mouse urine generalization to LLC mouse urine (Figure 1C-iv); LLC-trained mouse urine generalization to LKR mouse urine (Figure 1C-v).

In light of these challenges, we elected to pursue a more highly controlled animal model approach [20], [21] where many of the variables that make patient work so difficult can be controlled (Figure 1A). Our strategy was to first demonstrate that mice can be trained to discriminate urine samples from mice with tumors from control mice by odor alone. Once we had established this was possible, we then employed metabolic profiling (solid-phase-microextraction, followed by gas chromatography coupled with mass spectrometry) to show we could identify specific patterns of volatiles in urine that could distinguish tumor-bearing mice from control animals.

## Results

### Mouse Models of Lung Cancer

Lung tumors derived from mouse cell lines have similarities in morphology, histopathology, and molecular characteristics with human lung adenocarcinomas and can serve as useful first models [22]. We used two mouse lung cancer cell lines, LKR that was derived from a transgenic animal expressing mutated Kras and LLC, the Lewis lung cell carcinoma which arose spontaneously. Tumors were induced by inoculating each of these cell lines into different groups of mice (control mice were injected with the vehicle, saline, on the same schedule). The tumor growth curves for these two cell lines showed similar patterns (Figure 1B). Based on tumor growth curves, we collected urine samples that spanned stages of tumor growth for bioassay and for later chemical analyses.

### Olfactory Detection of Urinary Odor

We trained sensor mice (see methods) to discriminate between the odors of mouse urine samples collected from LKR-injected mice with large tumors (Days 25–37 post cell injection) compared to genetically identical control mice without tumors. When this was successful, we tested to determine whether this learning generalized to earlier stages of tumor development. As shown in Figure 1C-i, the trained mice successfully distinguished between urines collected from mice with tumors at 25–37, 15–20 and 9–14 days post injection but did not generalize to tumors at very early stages (Days 1–7). Next, we further trained these same sensor mice using urines collected on Days 15–24 post injection. Although these mice generalized this training to novel samples collected from mice with tumors the same size, they did not do so for urines collected on Days 9–14 or 1–7 (Figure 1C-ii). Control experiments verified that trained mice did not distinguish between injected and uninjected mice prior to injections, demonstrating that there was no bias in the original mouse urine or the Y-maze apparatus.

To investigate the generality of this result, we trained a separate group of sensor mice to discriminate urines of mice with- and without LLC-induced tumors. The pattern of results was almost identical to that with LKR-induced tumors (Figure 1C-iii).

We next asked whether the odors associated with the LKR and LLC tumors were perceptually similar, by testing the trained mice on urine samples collected from tumor-bearing vs. control mice from the animal model different from the one that they had been trained on. That is, we asked whether mice trained to discriminate urines of mice with and without LKR-induced tumors would recognize (generalize this response to) LLC tumor-bearing mice and vice versa. The answer was in the affirmative (Figure 1C-vi and -v). These results show that tumors induced by these cancer cell lines produce common (although not identical; see below) volatile biomarkers that can be recognized by the olfactory systems of mice.

### Characterization of Urinary Volatile Compounds

We next characterized the nature of chemical variation distinguishing mice with the tumors from those without by analyzing urinary volatile compounds with solid-phase-microextraction, followed by gas chromatography coupled with mass spectrometry. From the typical total ion chromatograms (TICs) a large diverse set of peaks could be distinguished (Figure S1). Forty seven peaks were selected for identification from the TICs based on their having sufficiently large peak heights and non-overlapping TICs as determined by visual inspection. As can be seen in Table 1 and S1, the peaks were comprised of a variety of chemical structures and are potentially involved in several biological functions, for example in pheromonal communication (2-heptanone, 3,4-dehydro-*exo*-brevicomin and 2-*sec*-butyl-4,5-dihydrothiazole, 6-hydroxy-6-methyl-3-heptanone, *β*-farnesene [23]). Also identified were compounds previously reported in human urine (nitromethane, dimethyl sulfone, *o*-toluidine, 2-ethylhexanoic acid [24]).

**Table 1:** Selected peaks and their identifications.

No.	Cell lines with p<0.0001	Compounds
2,4,5,6	LKR and LLC	5,5-dimethyl-2-ethyltetrahydrofuran-2-ol
7	LLC	nitromethane
8	LKR	2-heptanone
11	LLC	unkown 2 compounds
13	LKR	5-hepten-2-one (E or Z)
18	LKR and LLC	2-acetyl-1-pyrroline
19	LKR and LLC	2-isopropyl-4,5-dihydrothiazole
22	LKR and LLC	2-*sec*-butyl-4,5-dihydrothiazole
27	LLC	6-hydroxy-6-methyl-3-heptanone
33	LKR	*o*-toluidine
37	LLC	2-ethyl hexanoic acid
45	LKR	*N*-phenyl formamide

**Figure 2:**
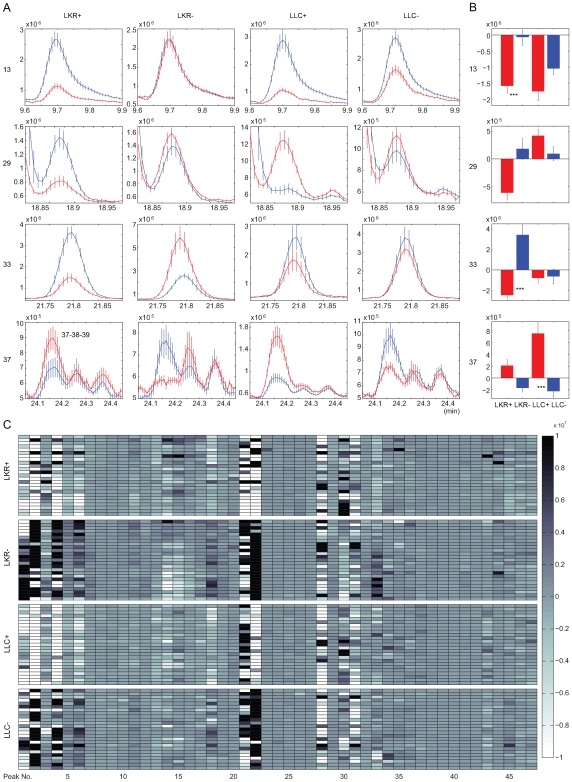
Comparison of selected peaks. (A) Comparison between early stage and late stage of 4 illustrative peaks selected from 47 peaks analyzed. Vertical axis indicates intensity (amount) of TIC; vertical lines around mean indicate SEM at each sampling point. Blue represents early stage whereas red represents late stage. Horizontal axis indicates retention time. (B) Bar plot of intensity of 4 illustrative peaks selected from the 47 peaks analyzed. Mean peak intensity is plotted at each peak. Red bars represent tumor groups; blue bars represent control groups. A pale blue background indicates a significant difference at *P*<0.0001 between tumor and control groups. (C) Raw intensity of 47 analyzed peaks obtained by subtracting the early period from the later period (n = 25 for each of the 4 groups). Darker grey means the peak increased following tumor development whereas the lighter grey means the peak decreased following tumor development.

We next used quantitative analyses of these 47 peaks to determine if mice with and without experimentally-induced tumors could be distinguished. Variation in the raw peak heights clearly showed differences in the relative amounts of various compounds based on the presence or absence of tumor and cancer types (Figure 2A and Figure S2). We observed relatively consistent changes for many peaks and for both tumor groups with the most common pattern being a decreased production (down-regulation) in the tumor groups and either an increased production (up-regulation) or negligible change in the placebo groups (Figure S3). For example, peak 13 (5-hepeten-2-one) was down-regulated dramatically as a consequence of tumor presence (Figure 2B). Thus, an overall down-regulation of volatile compounds may be a common feature of tumor growth. However, there were other patterns of change for a minority of volatiles compounds. For example, production of peak 37 (2-ethyl hexanoic acid) was elevated in both tumor groups. Changes in other peaks depended on cancer types (and/or mouse strain). Peak 29 (Acetophenone) was down-regulation in the LKR-tumor group and up-regulation in LLC-tumor group whereas peak 33 was down-regulation only in LKR tumor group. The image array plot (Figure 2C) clearly shows the overall differential effects of tumor growth.

### Discrimination of Tumor and Placebo Groups

**Figure 3:**
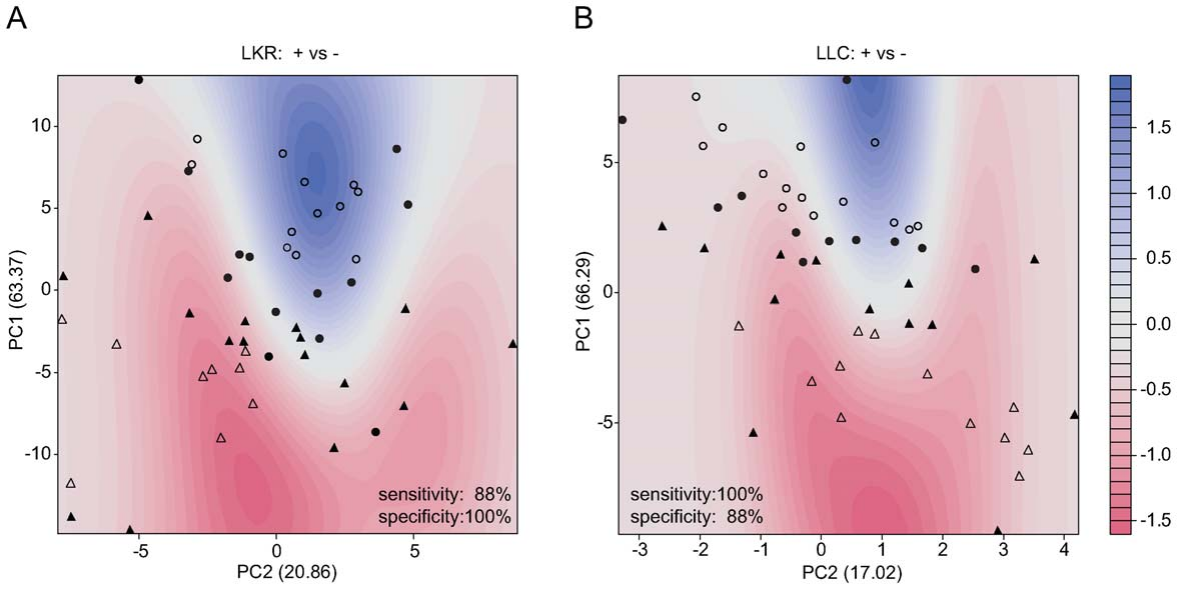
Separation of tumor and placebo groups based on PCA scores using SVM. Separation of tumor and placebo groups by Principle Components Analysis (PCA) and its boundary determination by Support Vector Machine (SVM) are shown in A (LKR) and B (LLC). Circles represent individuals of tumor groups and triangles represent individuals of placebo groups (Support vectors: solid circles and triangles). The background contour color, ranging from red to blue, indicates the class probability for different regions of the plane.

We next proceeded to metabolomic profiling to statistically discriminate between groups and to identify characteristic peaks. To this end, we combined two different approaches: principal component analysis (PCA) and support vector machine (SVM). The first, PCA, allows the structure in a dataset to dictate the separation of samples into clusters based on overall similarity in peak values without prior knowledge of sample identity. Plots of PCA scores calculated from the normalized values of the 47 peaks showed a distinctive separation of the chemical profile between tumor groups and placebo groups in both cancer cell lines (Figure 3A and 3B). Second, a supervised machine-learning approach based on the SVM was employed to determine the boundary between tumor groups and placebo groups. This algorithm considered the first two principal components, PC1 and PC2, to create descriptions of samples in this high-dimensional space, and then defined a hyperplane that best separates samples from the two classes. The SVM classifier successfully separated the samples into tumor and placebo categories (displayed in the fine contour with color of blue to red in Figure 3A and 3B). The SVM successfully classified most individuals giving a classification accuracy of 94% with a sensitivity of 88 % and specificity of 100% (LKR) and an accuracy of 94% with a sensitivity of 100% and specificity of 88% (LLC). Notably, only 3 of the 50 individual mice in our test set were misclassified. Thus, the selected peaks contain chemical features distinguishing tumor from placebo mice.

### Cross Validation and Essential Combination of Peaks

To validate these analyses, we employed a 10-fold cross validation method by using all 25 samples. For further analysis, we selected 11 peaks from the original 47 peaks that differed between tumor and placebo groups with a *P*<0.0001 (Table 1). We trained the SVM classifier by applying all logically possible combinations without repetitions from these 11 peaks for each of the two model systems (LKR and LLC). The generalization performance of the SVM classifiers employing different sets of peak clusters was illustrated in receiver operating characteristic (ROC) space. No single peak successfully classified with an accuracy of greater than 95%. However, classification with several pairs of peaks resulted in an accuracy of up to 98±2% for LKR and 100% for LLC (Table 2-i,-ii and Table S2, Figure S4), confirming that better generalization relies upon a combination of peaks. In further analyses (data not shown) we found that SVM had superior performance to Fisher Discriminant Analysis, which used unsupervised learning methods. Thus, characteristic peak clusters can reliably differentiate tumor groups from placebo groups and may have diagnostic potential.

**Table 2:** Summary of highest scores of SVM classifiers.

Summary of highest scores of SVM classifiers	
Numbers of Peaks	Accuracy		Sensitivity		Specificity	
**i) LKR ten-fold CV**	**mean**	**sem**	**mean**	**sem**	**mean**	**sem**
Nos. 7, 13, 22	98	2	97.5	2.5	100	0
Nos. 8, 13, 18, 19, 22, 45	98	2	100	0	95	5
**ii) LLC ten-fold CV**						
Nos. 5, 11, 19, 37 (or 2, 4, 6, 19, 37)	100	0	100	0	100	0
**iii) LKR to LKR (training, 13 samples; test, 12 samples)**						
Nos. 7, 8, 13	95	5.6	91.67	0	98.33	1.11
Nos. 13, 33, 45 (or 8, 13, 33, 45)	95	8.3	90	1.67	100	0
**iv) LKR to LLC (training, 13 samples; test, 12 samples)**						
Nos. 13, 22, 33, 45 (or 7, 13, 19, 22, 33, 45)	98.33	1.67	100	0	96.67	3.33
**v) LLC to LLC (training, 13 samples; test, 12 samples)**						
Nos. 2, 6, 19, 37 (or 4, 5, 19, 37)	100	0	100	0	100	0
**vi) LLC to LKR (training, 13 samples; test, 12 samples)**						
Nos. 5, 11, 22, 27, 37	91.25	4.2	91.67	0	90.83	8.3

To assess the generalization power of the peak clusters to a novel group, we created independent training sets (13 of the 25 samples) and test sets (remained 12 samples). The SVM classifiers trained with the 11 selected peak clusters of the training set generated a best combination of peaks having accuracies of 95% for LKR and of 100% for LLC to test sets (Table 2-iii,-v and Table S3, Figure S5).

Although LKR and LLC cell lines are different model systems and they were injected into different inbred mouse strains (which themselves likely differed in body odors), our behavioral studies suggested that they shared common odors indicative of the presence or absence of tumors. This was also found to be the case in the metabolomic analyses. The group of peaks (the cluster) that best predicted LLC status from LKR data as determined by SVM (Table 2-iv) had an accuracy of 98%. Conversely, the group of peaks that best predicted LKR from LLC (Table 2-vi) had an accuracy of 91%. Only one peak (# 22; see Table 2-iv,-vi) was common to these two sets of predictive clusters. Classification by other peak clusters also generated high diagnostic accuracy (95%) with substantial diagnostic potential (Figure S6).

### Interactive Effect of Tumors and Cell Lines

**Figure 4:**
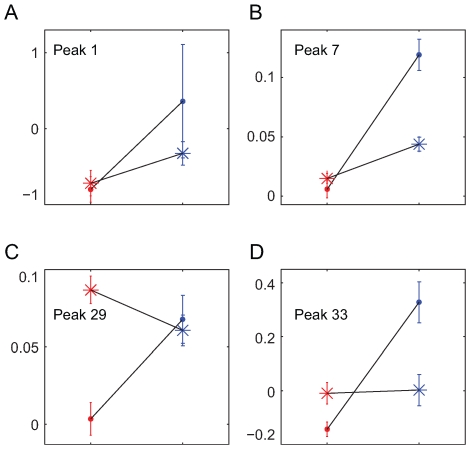
Interactions between tumor type and tumor stage. Normalized intensity (on the vertical axis) of the four peaks (A−D) in which a two-way ANOVA indicated significant (*P*<0.002) interactions indicating differentiation between the two tumor models. The horizontal axis of each of the 4 panels (A−D) indicates the two stages, early - prior to significant tumor development on the left and later - after development of significant tumor size. Red: tumor, Blue: placebo, Circle: LKR, Star: LLC.

Even though there were commonalities between the two tumor models, further statistical analyses also demonstrated that the effects of the two tumor models on metabolic profiles were not identical. The interactions between two different cell lines (LKR and LLC) and tumor vs. placebo was analyzed with 2-way analyses of variance (tumor and placebo groups for each of the two tumor models) for each of the 47 peaks (Figure 4 and S7). A significant interaction indicates tumor specificity. Of the 47 separate analyses, the interaction w'as significant (*P*<0.05) in 11 cases (Table S3). To control for false positives due to testing 47 peaks, we restricted consideration to 4 peaks (No. 1, 7, 29, and 33) with *P*<0.002. These interactions are illustrated in Figure 4 where, for example, peak 29 shows no difference between tumor and placebo (*P* = 0.0387) but a large difference between tumor models (*P* = 0.0002). There is sufficient specificity to distinguish between the volatile profiles of the two tumor types.

## Discussion

Identification of volatile biomarkers in urine for disease diagnosis is an area of great promise, however it is based on limited prior human research. The data in this paper are consistent with the hypothesis that diagnostically useful volatile compounds are produced in patients with lung cancer and secreted into the urine, thus providing support for this diagnostic approach in the context of lung cancer.

Specifically, our studies showed that animal olfaction in species like the mouse (that has a sensitive olfactory system [25], [26]), can have diagnostic success in detecting lung cancer signatures in urine. More importantly, we were able to mimic this ability using bioanalytic techniques. This suggests it will be possible to create a biomimetic sensor based on the knowledge of olfactory system for screening diagnostic odorants that could be practical for widespread applications [27], [28], [29], [30]. Indeed, genetically engineered yeast expressing an olfactory receptor and its signal transduction system have been shown to be capable of detecting 2,4-dinitrotoluene, a compound diagnostic of explosives [31]. Artificial olfaction with a polymer epithelium and model glomeruli could detect odorants thereby mimicking a biological olfactory system [32]. Ultimately, such sensors could lead to the development of commercially available test kits. However, it also appears that metabolic profiling (solid-phase-microextraction, followed by gas chromatography coupled with mass spectrometry) is a viable alternative that should be further explored.

The metabolic origin of many of the diagnostic biomarkers we identified is not known and we could not identify common chemical features. Instead, they had their origins in either a variety of endogenous biochemical pathways or from environmental (exogenous) sources. These latter compounds (e.g. *o*-toluidine, and 2-ethylhexanoic acid) are unlikely to be diagnostically useful. Among the endogenous metabolites, 2-heptanone, a pheromone, is reported to increase in concentration in stressed rats, and has been observed in human urine [33]. 6-hydroxy-6-methyl-3-heptanone has also been previously identified in mouse urine although we can find no report of this compound in human urine. The observed variation of ketones as a function of tumor growth suggests that ketogenesic pathways may be involved in these models of lung cancer. Further research would be required to determine which of these diagnostic metabolites are of tumor origin and which originate from normal metabolic processes and are either down- or up-regulated by the tumors.

The common down-regulation we observed following tumor development in many compounds is noteworthy. Most biomarkers reported by other investigators have been up-regulated. One explanation for these different findings may reside with detection strategies employed by others to identify biomarkers. In some investigations there may be a bias toward a search for novel (and therefore up-regulated) biomarker compounds whereas our methods had no such bias. Another possibility is that this frequent down-regulation results from complex effects of the tumor on the animal's metabolism.

Although SVM found clusters of peaks that predicted between the two models of cancer (LKR to LLC and vice versa: Table 2) with high accuracy, the clusters were mainly different for prediction in the two directions. This result appears to be inconsistent with the animal training studies that indicated that mice trained to discriminate in one of the tumor models generalized this learned response without further training to the other model. This implies that there should be a set of volatile compounds (odorants) common to the two models that differentiate tumor from non-tumor mice. One likely explanation for this apparent anomaly is that the combinations of volatile components that we have identified with SVM classifiers are not the same ones that the mice are cueing in on during training and testing; perhaps there are other components in common with the two models that we have not yet identified. If this is the case, one of the next challenges will be to identify these novel biomarkers. Alternatively, we note that there was one compound that was common to prediction in both directions (#22) and we cannot exclude the possibility that it was this compound the mice used to make the distinction in both cases.

An important consideration for any practical diagnostic tool is its ability to discriminate among different types of disease. Although the two models of lung cancer clearly have similarities in volatile profiles, they also have sufficient differences that they can be discriminated in metabolomic analyses (Figure 4). This ability to discriminate between closely related mouse models of lung cancer implies that specific cancer types may be amenable to diagnostic differentiation through analyses of volatile profiles as illustrated in the current research.

Future work with animal models could proceed along three convergent lines. First, it is important to monitor the developmental changes in markers at earlier stages of tumor development. Not only is this relevant to determining how early diagnostic markers can be detected but it could throw light on potential mechanisms underlying changes in volatiles as a consequence of the tumor progression. Second, a variety of different tumor types should be investigated in addition to the two closely related ones described here. This could also provide important clues as to mechanism. Third, in vitro studies on tumor cells will be crucial in understanding mechanisms.

In summary, we were able for the first time to identify volatile chemical signatures in urine of mouse models of lung cancer using rigorous experimental behavioral and analytic techniques. The importance of this study is that it establishes the feasibility of using urinary volatiles to detect lung cancer. The ability to easily collect and store urine samples will be a major advantage of this approach over analyzing volatile in exhaled breath. Although this study has raised many questions about the identity and source of the compounds detected in our mouse models, we are not planning to pursue this direction. Instead, we view this study as an important proof of principal for the value of studying urine volatiles using biochemical and bioinformatic techniques in the diagnosis of human lung cancer (and perhaps other cancers). Accordingly, we have begun clinical studies with human patients. These studies will address key questions about sensitivity, specificity, the size of tumors that can be accurately detected, the mechanisms underlying the observed changes in volatile profiles, the ability to generalize among different types of lung cancers, and the impact of current or former smoking.

## Materials and Methods

### Lung Cancer Cell lines

The Kras-induced murine lung cancer (LKR) and Lewis lung cell carcinoma (LLC) cell lines were purchased from the ATCC (American Type Culture Collection, Manassas, VA). LKR cells were derived from explants of a pulmonary tumor from an activated K-*ras*G12D mutant mouse grown in Dr. Tyler Jacks' Laboratory at M.I.T. [34]. Cells were cultured and maintained in high glucose DMEM (Mediatech, Washington, DC) supplemented with 10% fetal bovine serum (Georgia Biotechnology, Atlanta, GA), 2 mmol/L glutamine, and 1% penicillin/streptomycin [35].

### Flank Tumor Model Mice of LKR and LLC Cell Lines

To create peripheral tumors 100 µL of 1×10^6^ LKR cells were injected in the flanks of male C57BL/6J×129P3/J F1 hybrid mice (group 1: n = 27; group 2: n = 22: LKR tumor groups). For control purposes, 100 µL of saline was injected in individuals of LKR-placebo group (n = 26). In the second model, 100 µL of 1×10^6^ LLC cells were injected in the flanks of 8-week-old male C57BL/6J mice (n = 32, LLC-tumor group) and for control mice 100 µL of saline was injected into same strain individuals of the LLC-placebo group (n = 31). Tumors were measured weekly beginning 1 week after injection with a digital caliper and volumes were estimated using the formula 3.14×[largest diameter×(perpendicular diameter)^2^]/6. The measured points were fitted with a cubic function implemented in MATLAB. All mice used in these experiments were maintained on a 12∶12 h light∶dark cycle. All mice were given food and water ad libitum throughout the experimental period. Tumor-injected mice were euthanized when it appeared that the tumor burden induced discomfort. The mice were cared for in accordance with the Guide for the Care and Use of Laboratory Animals and the experimental protocols were previously approved by the Institutional Animal Care and Use Committee in Monell Chemical Senses Center (Approval number: 1113p).

### Urine Collection for Bioassay and Chemical Analysis

Voided mouse urine was collected individually once a day, 5 days per week by gentle abdominal pressure into a sterile glass tube [36]. Immediately, urine samples were frozen at −20°C and retained until needed for experiments.

### Sensor Mice and Behavior Assessment in the Y-Maze Apparatus

Mice (Male C57BL/6, n = 6; female C57BL/6 H-2^b^, n = 1) were trained in a Y-maze apparatus to discriminate between urines of tumor bearing mice compared with placebo (non-tumor bearing) mice [37]. Briefly, the two arms of the maze were scented by air currents conducted through chambers containing freshly-thawed urine (0.3–0.4 ml placed in 3.5-cm-diameter Petri dishes). For training and testing in the Y-maze, gates were raised and lowered in a timed sequence of up to 48 consecutive trials, paired urine samples (tumor vs. placebo) being changed for each trial. During the training session, water-deprived sensor mice for 23hr were rewarded with a drop of water for each correct response. After successful training (>80% correct scores), unrewarded (generalization) trials were interspersed at an average frequency of one in four to accustom the mice to occasional absence of reward.

For one group of trained mice (n = 7), training samples consisted of urine collected 25–37 days after either LKR cell injections (LKR tumor mice) or placebo injection (LKR-placebo mice) (Figure 1B). Four mice were reinforced in the Y-maze when they chose the urine scent of LKR tumor mice over LKR placebo mice whereas the other 3 trained mice were reinforced for the opposite choice. Within 14 days, all mice were responding correctly at >80% accuracy. At this point generalization trials with novel samples were introduced (see next paragraph). A second group of trained mice (female C57BL/6, n = 3; female C57BL/6 H-2^k^, n = 1) were trained on LLC tumor vs. LLC placebo urine samples collected on days 17–26 post-cell or placebo injections. Here 2 mice were reinforced for LLC tumor and the other two were reinforced for LLC placebo.

Mice were then tested in generalization trials with novel urine samples (by blind testing) that were collected from LKR experimental (tumor bearing) and LKR placebo mice during days 2–7, 9–14, 15–20, and 25–37 and for LLC experimental and placebo mice during days 1–8, 9–16, and 17–26 (Figure 1B). To test for detection of urine odor changes at earlier stages of tumor development, LKR-trained mice were retrained on samples collected on days 15–24 of LKR and then were given a series of generalization tests. Additionally, LKR trained mice were given a series of generalization tests with LLC tumor vs. control samples (these animals never having been trained on LLC) and, reciprocally, LLC-trained mice were with LKR samples. The animals were maintained on a 12 hr light/dark cycle and tested during their light period.

### Extraction of Mouse Urinary Volatile Compounds by Solid-Phase-Microextraction

We chemically analyzed 25 samples of urines for each group at two time points: early stage (Days 1–3) and terminal stage (Days 34–40) of the LKR-tumor and -placebo groups and, in parallel, early stage (Days 1–3) and terminal stage (Days 24–27) of the LLC-tumor and -placebo groups. One hundred µl of mouse urine was placed in a 4-ml glass vial and the volatiles in the headspace were extracted for 30 min at 40°C using a Solid-Phase-Microextraction (SPME) fiber (2-cm long, 30 µm carboxen, 50 µm divinyl benzene, polydimethyl siloxane, Supelco Corp, Bellefonte, PA). No salt addition or pH adjustment was performed on the mouse urine.

### Gas Chromatography and Mass Spectrometry

The SPME fiber with absorbed volatile compounds was inserted into the injection port of Thermo-Finnigan Trace GC/MS (Thermo Electron, San Jose, CA) system and desorbed for 5 min at 230°C. The Trace GC/MS was equipped with a Stabilwax column (30 M×0.32 mm with 1.0 µ coating; Restek, Bellefonte, PA) which was used for separation and analysis of the desorbed volatiles. We employed the following chromatographic protocol for separation before MS analyses: 60°C for 4 min, then programmed at 6°C/min to 230°C with a 40-min hold at this final temperature. Column flow was constant at 2.5 ml/min. The injection port was held at 230°C. Operating parameters for the mass spectrometer were as follows: ion source temperature, 200°C, ionizing energy at 70 eV; scanning frequency was 2/s from m/z 41 to m/z 400. Peak identification was accomplished through manual interpretation of spectra and matching against the NIST'02 library and comparison with commercially available standard samples when available.

### Data Processing of Raw GC/MS Chromatogram

Raw GC/MS chromatograms were pre-processed using methods similar to those described elsewhere [38]. Briefly, components were detected simultaneously across all samples, quantified, log-transformed, and then normalized for differences in overall intensity levels with MATLAB. Forty seven total ion peaks from the total ion chromatogram were selected, all of which are shared by each animal in every group, and the heights were quantified. To eliminate the effect of the treatment term from the first day to last day that may occur in both experimental and control groups we defined the change in the production level of volatile compounds as: *Ri* = *tHi*–*eHi* (*i* = 1, 2,…, 47), where *tHi* denotes the maximum height of each peak at the terminal stage (i.e. days 25–37 for LKR and 17–26 for LLC while *eHi* denotes the maximum height of each peak at the early stage (Days 1–3). Thus, the subtracted value (*Ri*) represents “the term effect” for the placebo groups (LKR(–) and LLC(–)) and “the term plus the tumor effect” for tumor groups (LKR(+) and LLC(+)) (Figure 2B). The subtracted value was normalized for data processing.

### Two-Way Analysis of Variance

We used two-way analysis of variance to assess the effects of lung cancer and tumor development on relative subtracted value for each compound. The following statistical model [39] was fit separately for each compound to assess the effects of lung cancer and tumor development on relative subtracted value:
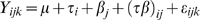
where 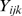 is normalized subtracted value, 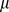 is the overall average, 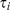 is the relative effect of tumor effect *i* (i = 1, 2 corresponding to tumor and placebo), 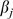 is the relative effect of lung cancer type *j* (j = 1, 2 corresponding to LKR and LLC), and 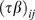 is an interaction effect describing the extent to which the lung cancer effect 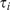 depends on strain effect 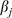. A significant interaction suggests that mice of different lung cancer types respond metabolically differently to tumor development. The random error term 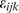 captures all other unexplained variation, and is assumed to have mean *0* and variance 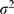.

### Principal Component Analysis and Boundary Determination by Support Vector Machine

Linear principal component analysis (PCA) was employed to examine the projection between two classes (e.g. LKR(+) vs. LKR(–)). First the first two principal components were plotted in the two dimensional space. The first principal component (PC1) accounts for as much of the variability in the data as possible, and each succeeding component accounts for as much of the remaining variability as possible.

For capturing the nonlinear boundaries between two classes (e.g. LKR(+) and (−)) as well as for understanding factors responsible for the group separation identified by PCA, we employed support vector machine (SVM) classifiers, which have been used with considerable success in a variety of fields including computational biology [40]. SVM finds an optimal hyperplane that separates two classes in high dimensional projected feature space. SVM maximizes the margin of separation between two classes to find a separating optimal hyperplane 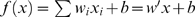, where 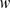 is the p-dimensional vector perpendicular to the hyperplane, *b* is the bias, *i* is the number of peaks. The SVM finds 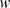 = (*w*_1_, *w*_2_, …, *w_p_*)*'* minimizing (1/2)||
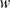
||^2^ subject to the constraints *y_i_*(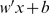)≥1, ∀*i* = 1, …, *n*, where *y_i_* = +1 *or* −1 depending on the class. When the training data are not linearly separable, SVM minimizes (1/2)||
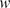
||^2^+*C* ∑*ξ_i_* subject to the constraints *y_i_*(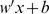
+*ξ_i_*)≥1, ∀*y_i_* = +1; *y_i_*(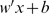
−*ξ_i_*)≥1, ∀*y_i_* = −1; *ξ_i_*≥0, ∀*i*. The *ξ_i_* is the slack variable. To realize this projection, we have used the Gaussian (also known as RBF) kernels of Kernlab package of R (http://www.r-project.org/), Spider MATLAB toolbox (http://www.kyb.tuebingen.mpg.de/bs/people/spider/) which implements linear function and Gaussian radial basis function: 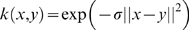, initialized radial basis function (RBF) dot with parameter 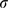 = 0.9.

The performance of the SVM classifiers was assessed by considering the number of correctly classified (true positives, tp; true negatives, tn) and incorrectly classified (false positives, fp; false negatives, fn) cases in the testing set. Sensitivity (se) was defined as the probability of a true positive, se = tp/(tp+fn); specificity (sp) as of a true negative, sp = tn/(tn+fp); and accuracy (ac) as the proportion of correct classifications, ac = (tp+tn)/(tp+fp+tn+fn). True positive rate (se) and false positive rate (1–sp) was plotted onto a receiver operator curve (ROC) space to perform diagnostic accuracy of SVM classifier. The error rate of the classifier is defined as the average number of misclassified samples, i.e. the sum of off-diagonal elements of the confusion matrix divided by the total number of objects.

### Ten-Fold Cross Validation and Generalization with Classifier

We used 10-fold cross validation to estimate diagnostic accuracy using 11 peaks that were selected based on the criterion that they were different between the tumor vs. control group (for LKR or LLC) at *P*<0.0001 (Table S1). We used 10-fold cross validation to estimate diagnostic accuracy using 11 peaks that were selected based on the criterion that they were different between the tumor vs. control group (for LKR or LLC) at *P*<0.0001 (Table S1).

In the first approach, the original 25 sample data sets from mice with tumors (e.g. LKR(+)) and the control mice (e.g. LKR(−)) were each randomly split into 10 sub-samples, each sub-sample consisting of 1–3 mice. The SVM classifier was then trained on 9 (10 minus 1) of the subgroups with a single subgroup being retained as the test sample. This cross-validation process was repeated 10 times (the folds), each of which has a different validation test group so that each sub-sample serves one time as a test sample. The resulting values for each of the folds were averaged to produce mean values for accuracy, sensitivity, and specificity (Figure S4, Table S2).

In a second approach, to determine a robust estimation of the generalization capability of classifiers for unknown samples, we randomly assigned 13 samples to a training set and left the remaining 12 samples for a test set (e.g. 13 LKR(+) and 13 LKR(−) were the training sets and 12 LKR(+) and 12 LKR(−) were the test sets). Using these sets, we determined the SVM classifiers using training sets made up of all logically possible combinations (combinations without repetition) of the selected 11 peaks (n = 2047 possible combinations). The classifiers were evaluated for accuracy, sensitivity, and specificity. In addition, to determine the predictive values across the two tumor cell lines (i.e. how predictive LKR is for LLC and vice versa), two additional tests were conducted. In one, the training sets were made up of LKR(+) and LKR(−) (n = 13 mice each) and the test sets were made up of LLC(+) and LLC(−) (n = 12 each). In the other this procedure was reversed. The calculated scores for these analyses were plotted in two dimensional spaces for sensitivity, and specificity (Figure S5 and S6) and are summarized in Table 2 and Table S2. We plotted onto a receiver operator curve (ROC) space to perform diagnostic accuracy of SVM classifier.

## Supporting Information

Figure S1 Image plot of total ion chromatogram (TICs). Total ion chromatograms of volatile compounds from urine samples collected during the early and late stages of the two tumor groups as well as parallel collections for the two placebo groups. TICs were pre-processed (see methods and reference). A typical TIC is shown in the top of (a) and the intensity is displayed as a colorized belt at the bottom of (a). All of TICs are displayed for LKR (b) and for LLC cell lines (c). The horizontal belt contains TICs from 25 animals. The horizontal axis represents retention time (later, far right).
(1.05 MB PDF)

Figure S2 Comparison between early stage and late stage of peaks. Forty-seven peaks were selected from the TICs for further analysis. Vertical axis indicates intensity (amount) of TIC; vertical lines around mean indicate SEM at each sampling point. Blue represents the early stage whereas red represents the late stage. Horizontal axis indicates retention time.
(3.99 MB PDF)

Figure S3 Bar plot of the intensity of the 47 peaks. Mean peak intensity is plotted for each peak. Red bars represent tumor groups whereas blue bars represent control groups. A pale blue background indicates a significant difference at P<0.0001 between tumor and control groups. It is possible to create a "bar code" representing each individual mouse.
(0.47 MB PDF)

Figure S4 Classification performance in ROC space of paired peaks. Each box (A-K) provides an overall visual representation of ROC obtained from the ten-fold cross validation of LKR. Red dots indicate mean sensitivity (vertical axis) and mean specify (horizontal axis, 1-specificity) for each classifier. The number of paired peaks are: A, 1; B, 2; C, 3; D, 4; E, 5; F, 6; G, 7; H, 8; I, 9; J, 10; K, 11. Each box (L–V) provides an overall visual representation of ROC obtained from the ten-fold cross validation of LLC. Red dots indicate mean sensitivity (vertical axis) and mean specify (horizontal axis, 1-specificity) for each classifier. The number of paired peaks are: L, 1; M, 2; N, 3; O, 4; P, 5; Q, 6; R, 7; S, 8; T, 9; U, 10; V, 11.
(0.64 MB PDF)

Figure S5 Generalization performance in ROC space of paired peaks. Each box (A–K) provides an overall visual representation of ROC obtained from generalization to the LKR test group by the LKR training group. Red dots indicate mean sensitivity (vertical axis) and mean specify (horizontal axis, 1-specificity) for each classifier. The number of paired peaks are: A, 1; B, 2; C, 3; D, 4; E, 5; F, 6; G, 7; H, 8; I, 9; J, 10; K, 11. Each box (L–V) provides an overall visual representation of ROC obtained from generalization to the LLC test group by the LLC training group. Red dots indicate mean sensitivity (vertical axis) and mean specify (horizontal axis, 1-specificity) for each classifier. The number of paired peaks are: L, 1; M, 2; N, 3; O, 4; P, 5; Q, 6; R, 7; S, 8; T, 9; U, 10; V, 11.
(0.67 MB PDF)

Figure S6 Generalization performance in ROC space of paired peaks. Each box (A–K) provides an overall visual representation of ROC obtained from generalization to the LLC test group by the LKR training group. Red dots indicate mean sensitivity (vertical axis) and mean specify (horizontal axis, 1-specificity) for each classifier. The number of paired peaks are: A, 1; B, 2; C, 3; D, 4; E, 5; F, 6; G, 7; H, 8; I, 9; J, 10; K, 11. Each box (L–V) provides an overall visual representation of ROC obtained from generalization to the LKR test group by the LLC training group. Red dots indicate mean sensitivity (vertical axis) and mean specify (horizontal axis, 1-specificity) for each classifier. The number of paired peaks are: L, 1; M, 2; N, 3; O, 4; P, 5; Q, 6; R, 7; S, 8; T, 9; U, 10; V, 11.
(0.70 MB PDF)

Figure S7 Regulatory factor determined by two-way ANOVA. Normalized intensity of subtracted peaks in a two-way ANOVA. Red: tumor, Blue: placebo, Circle: LKR, Star: LLC.
(0.41 MB PDF)

Table S1 (0.32 MB PDF)

Table S2 (0.31 MB PDF)

Table S3 (0.24 MB PDF)
