# Supplementary figures and images for: Urinary Volatile Compounds as Biomarkers for Lung Cancer: A Proof of Principle Study Using Odor Signatures in Mouse Models of Lung Cancer
Source: PLoS One. 2010 Jan 27;5(1):e8819. doi: 10.1371/journal.pone.0008819 (PMC2811722; doi:10.1371/journal.pone.0008819)

Figure S1 Image plot of total ion chromatograms

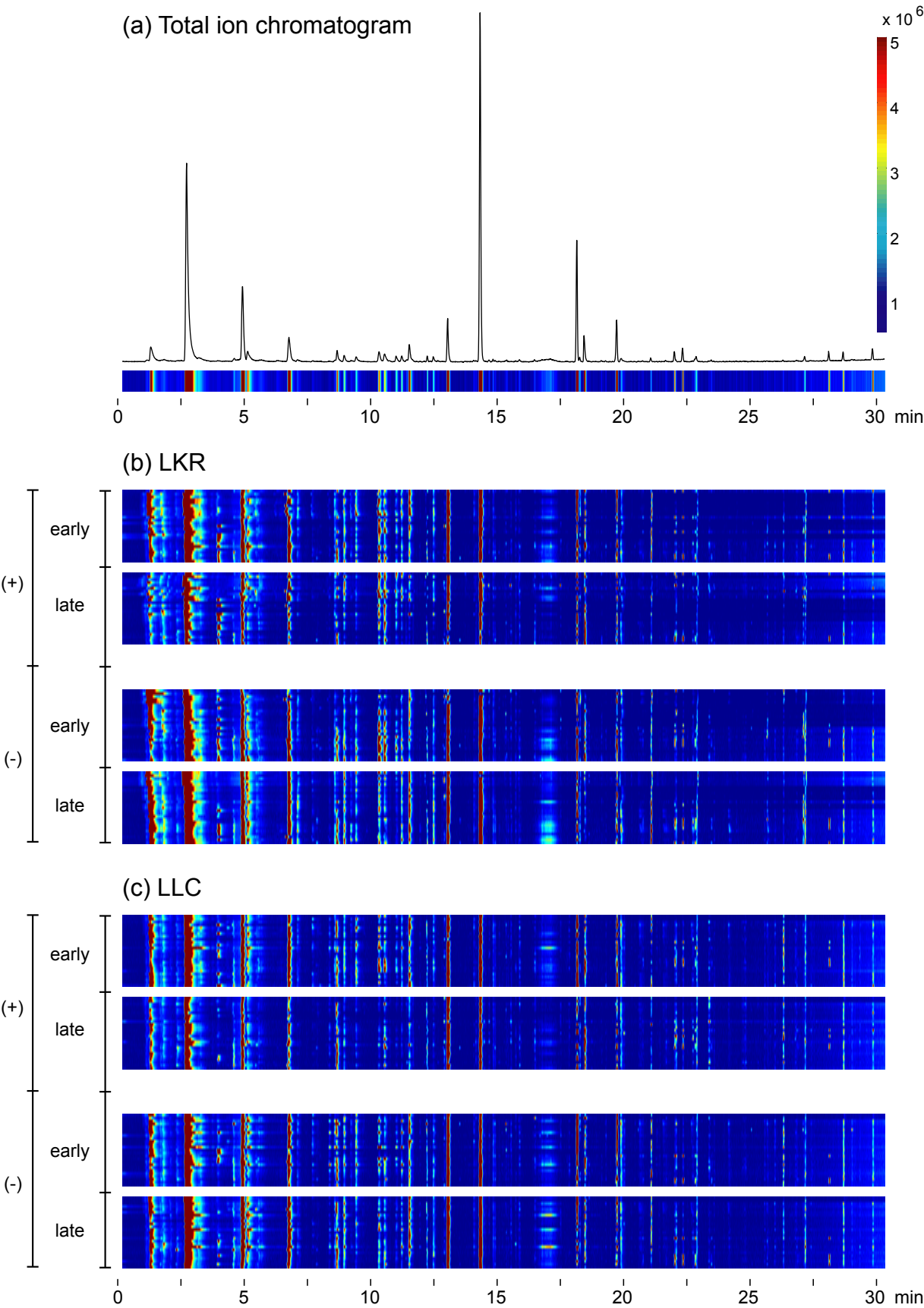

Supplement: Figure S1 — Image plot of total ion chromatogram (TICs). Total ion chromatograms of volatile compounds from urine samples collected during the early and late stages of the two tumor groups as well as parallel collections for the two placebo groups. TICs were pre-processed (see methods and reference). A typical TIC is shown in the top of (a) and the intensity is displayed as a colorized belt at the bottom of (a). All of TICs are displayed for LKR (b) and for LLC cell lines (c). The horizontal belt contains TICs from 25 animals. The horizontal axis represents retention time (later, far right). (1.05 MB PDF) [file pone.0008819.s001.pdf]

Figure S2

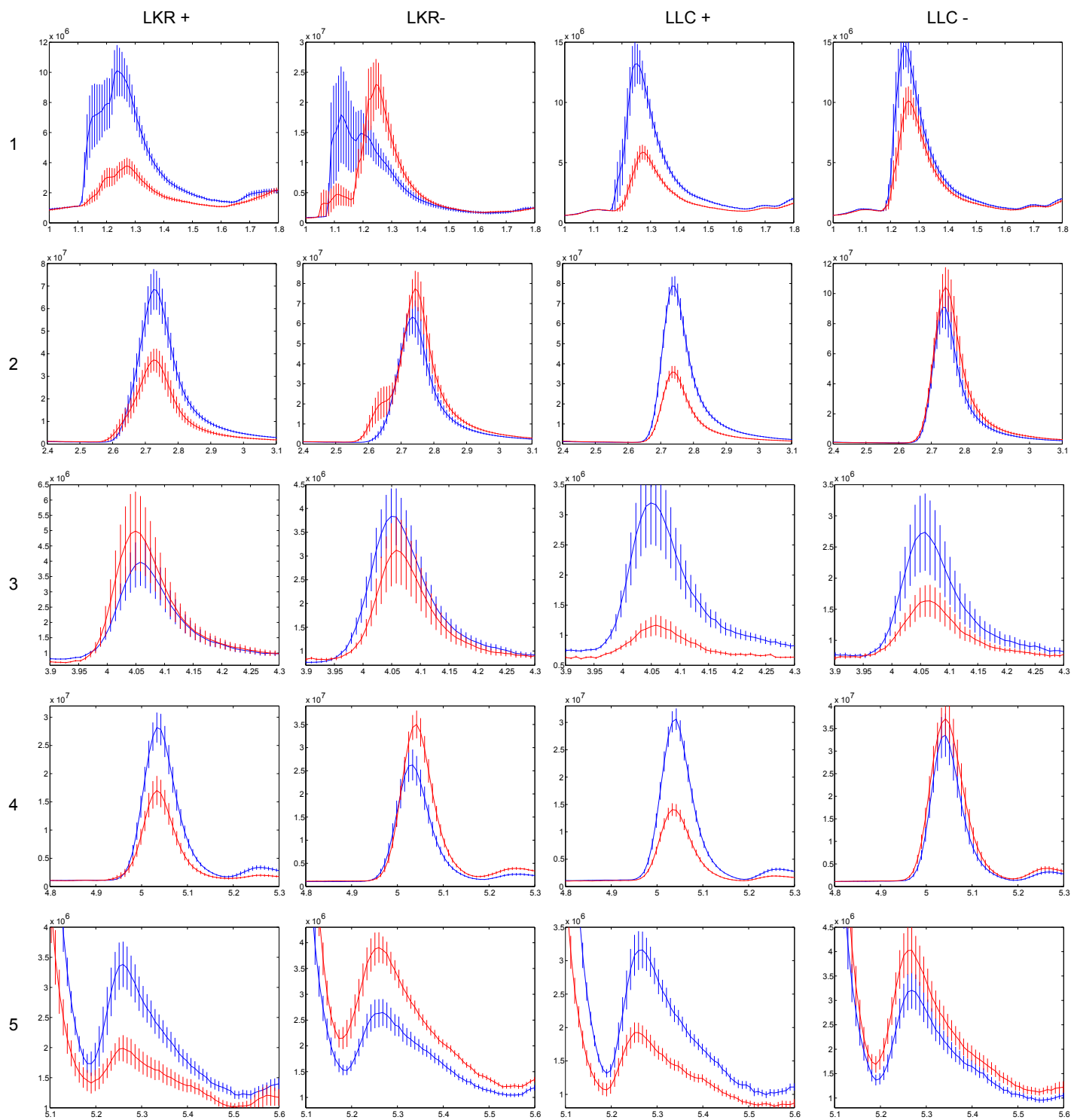

Blue: early stage; Red: late stage

Figure S2

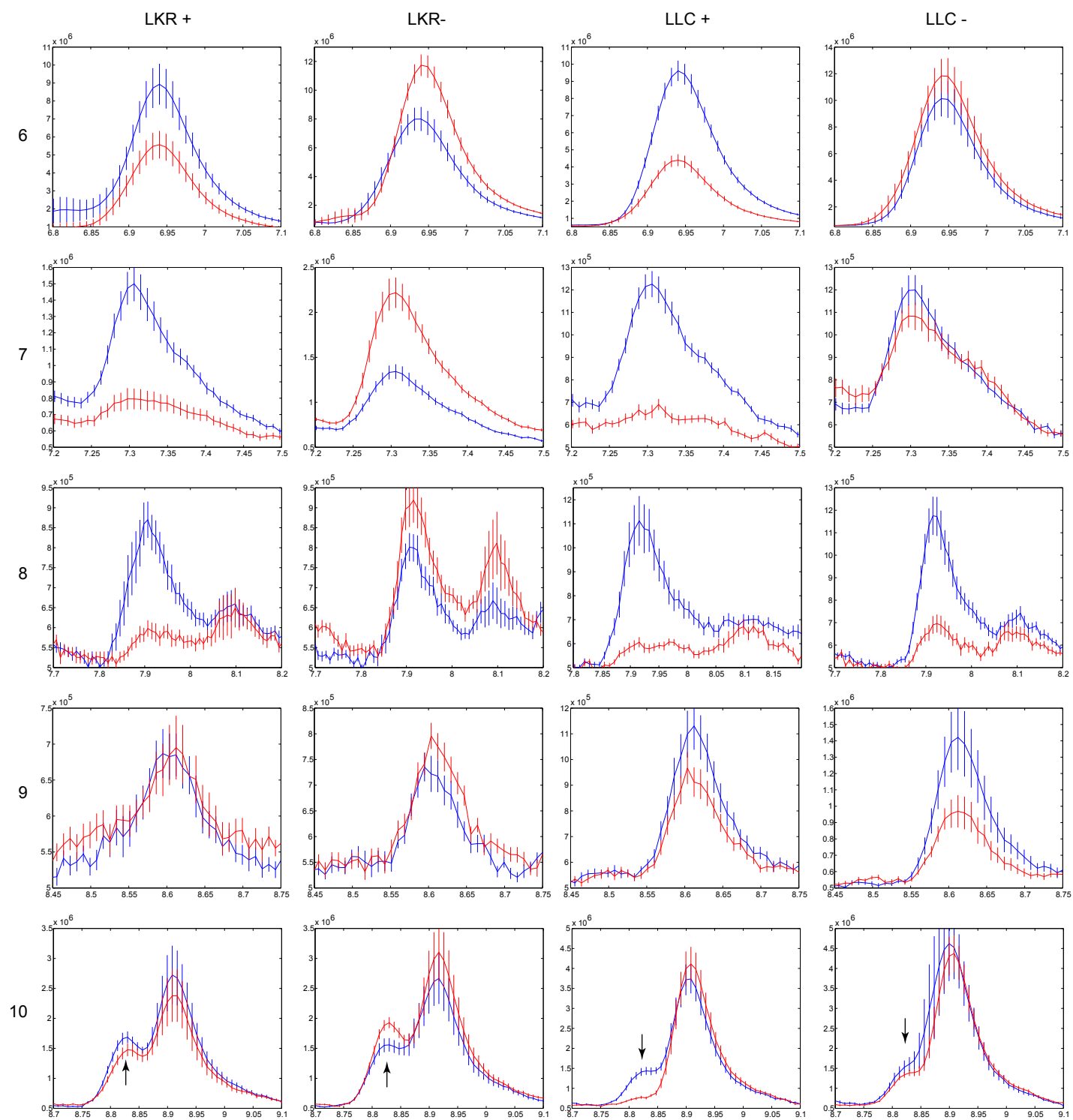

Figure S2

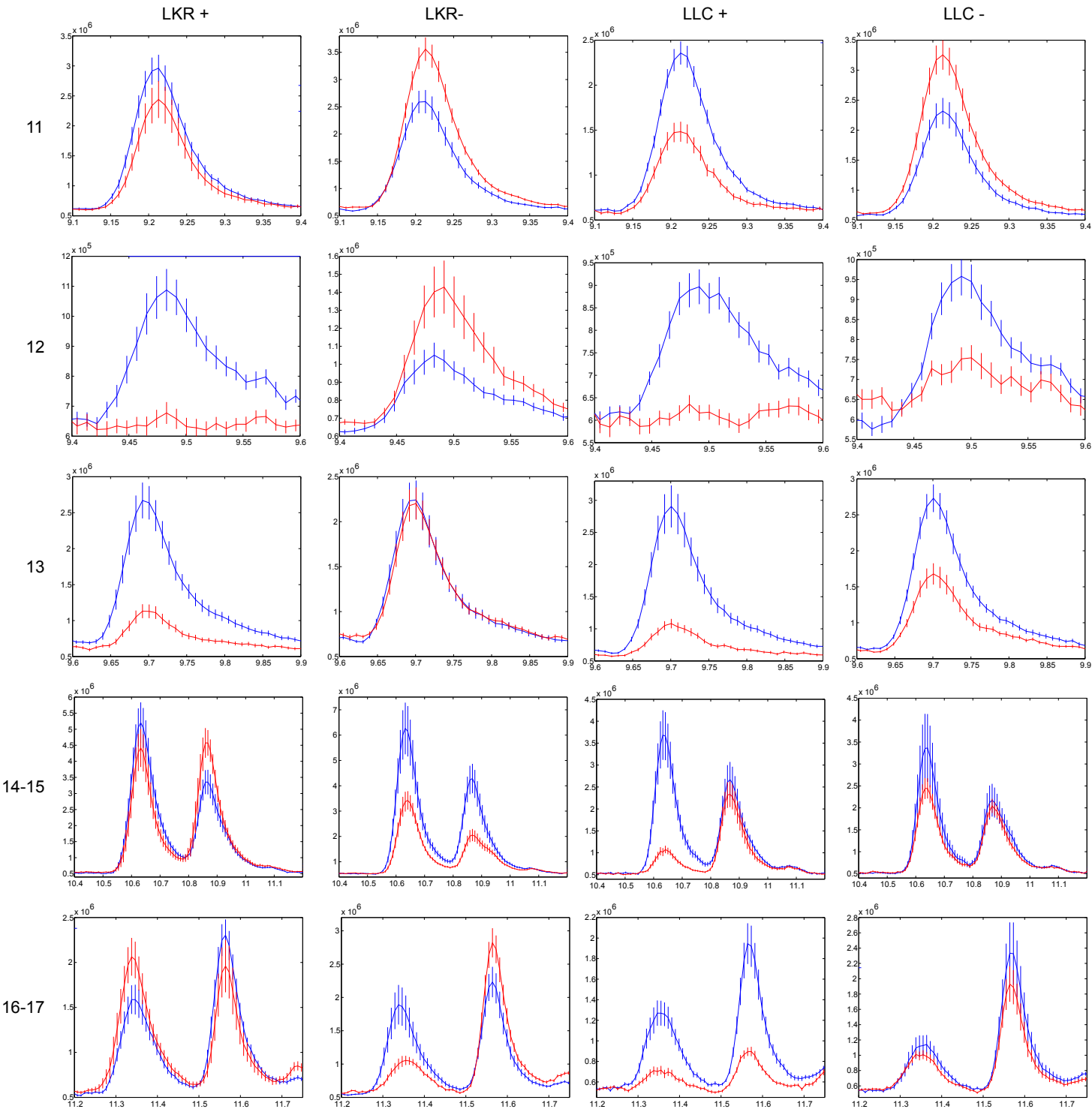

Figure S2

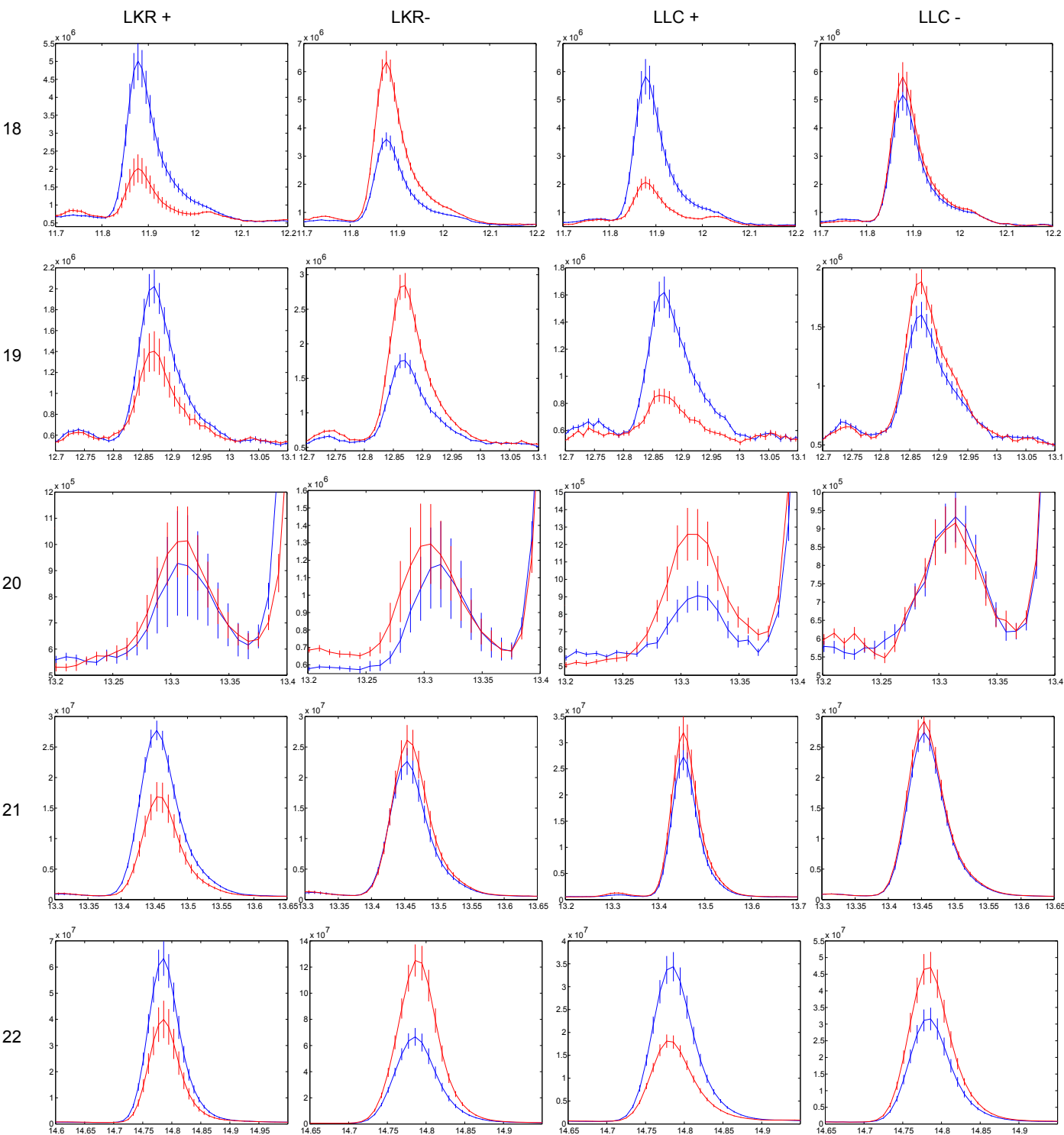

Figure S2

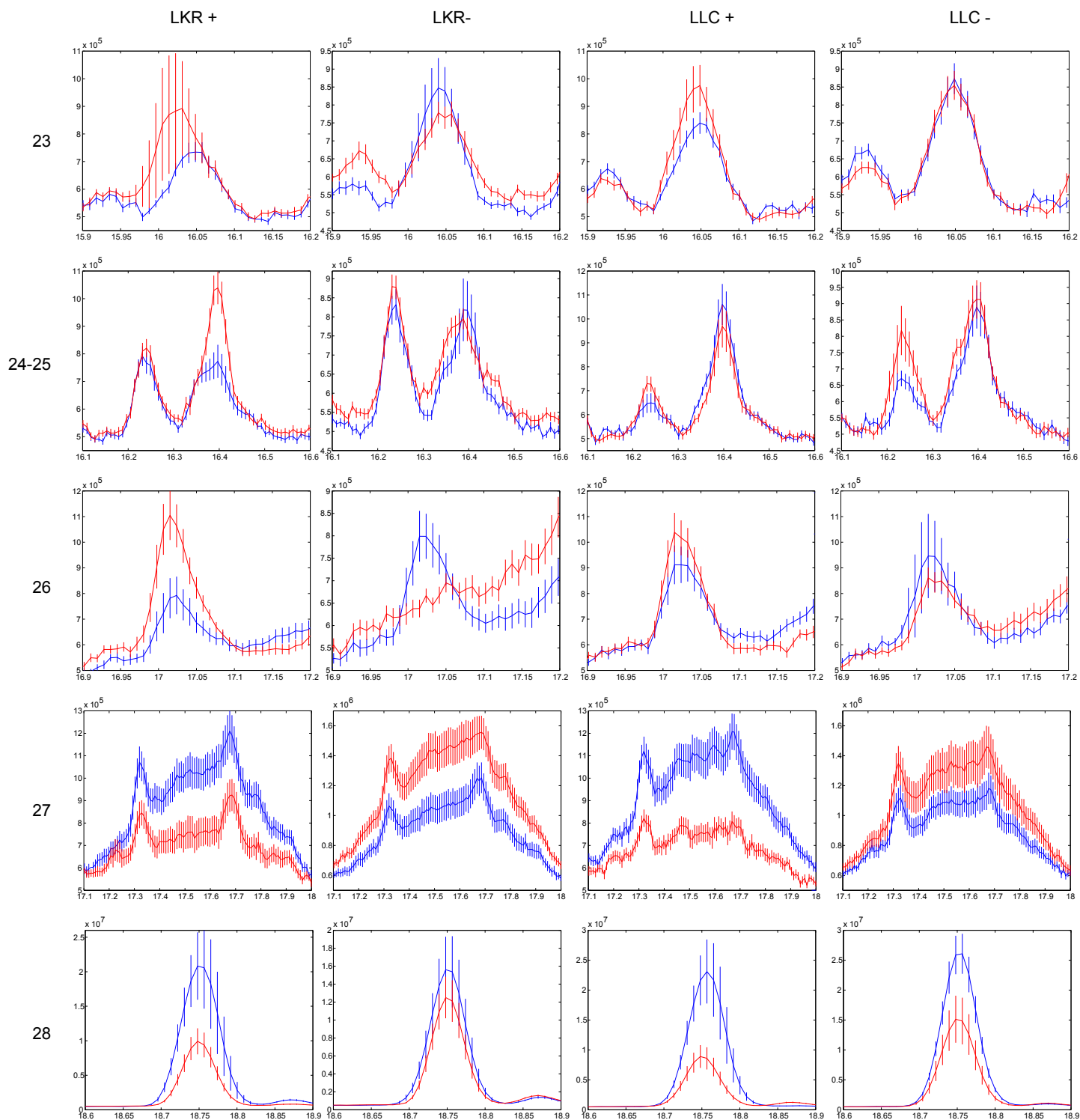

Fig. S2

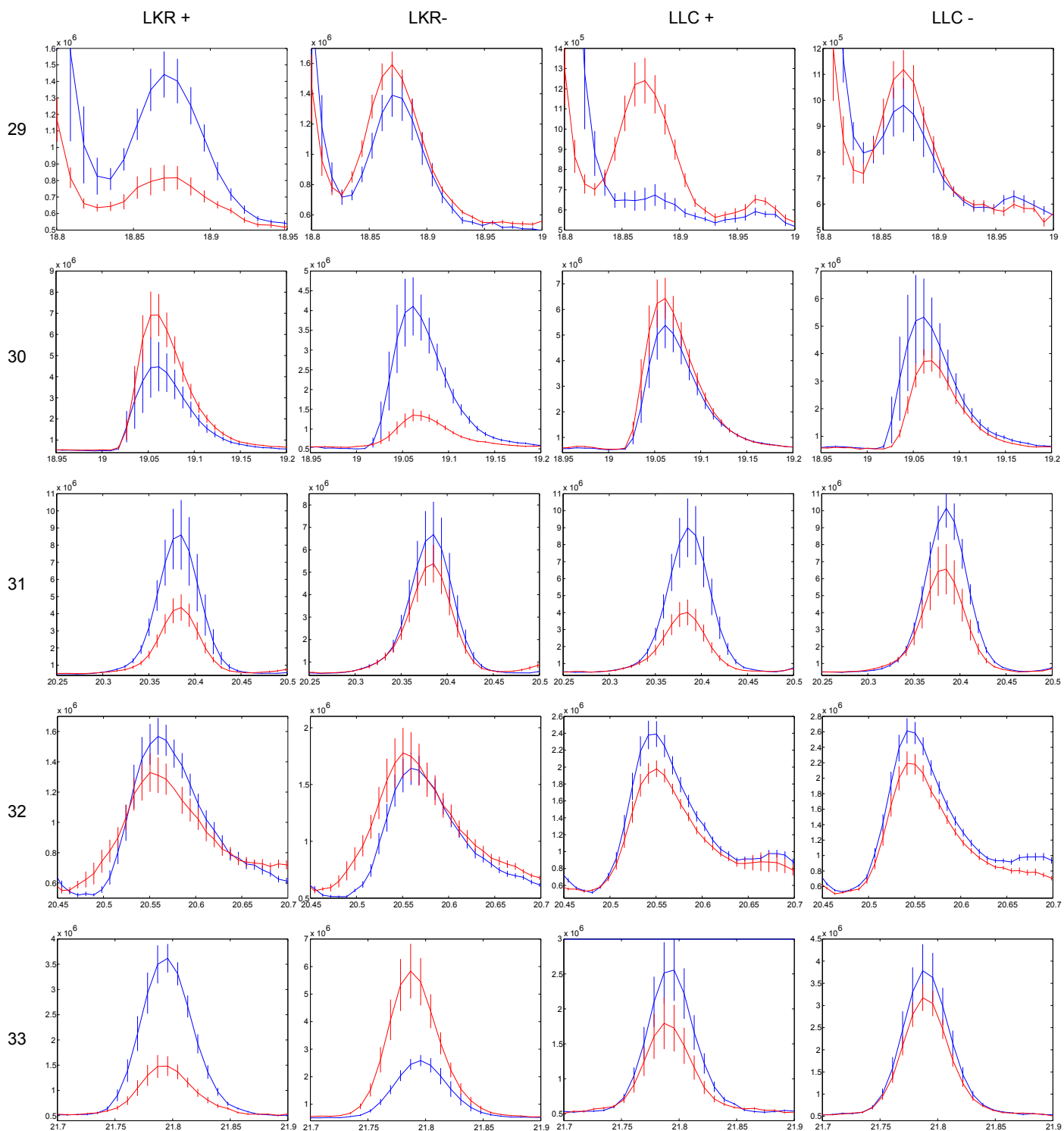

Figure S2

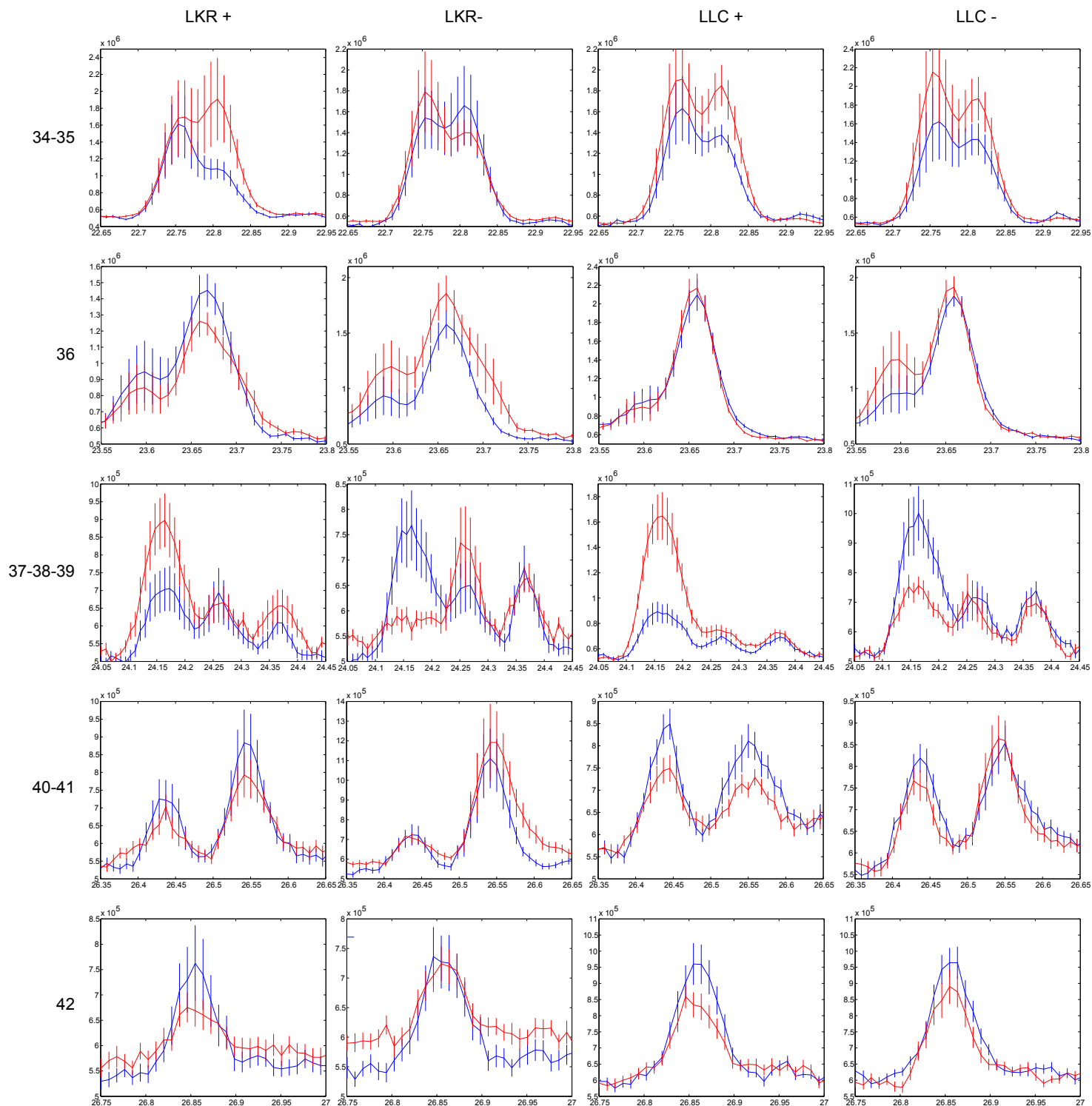

Figure S2

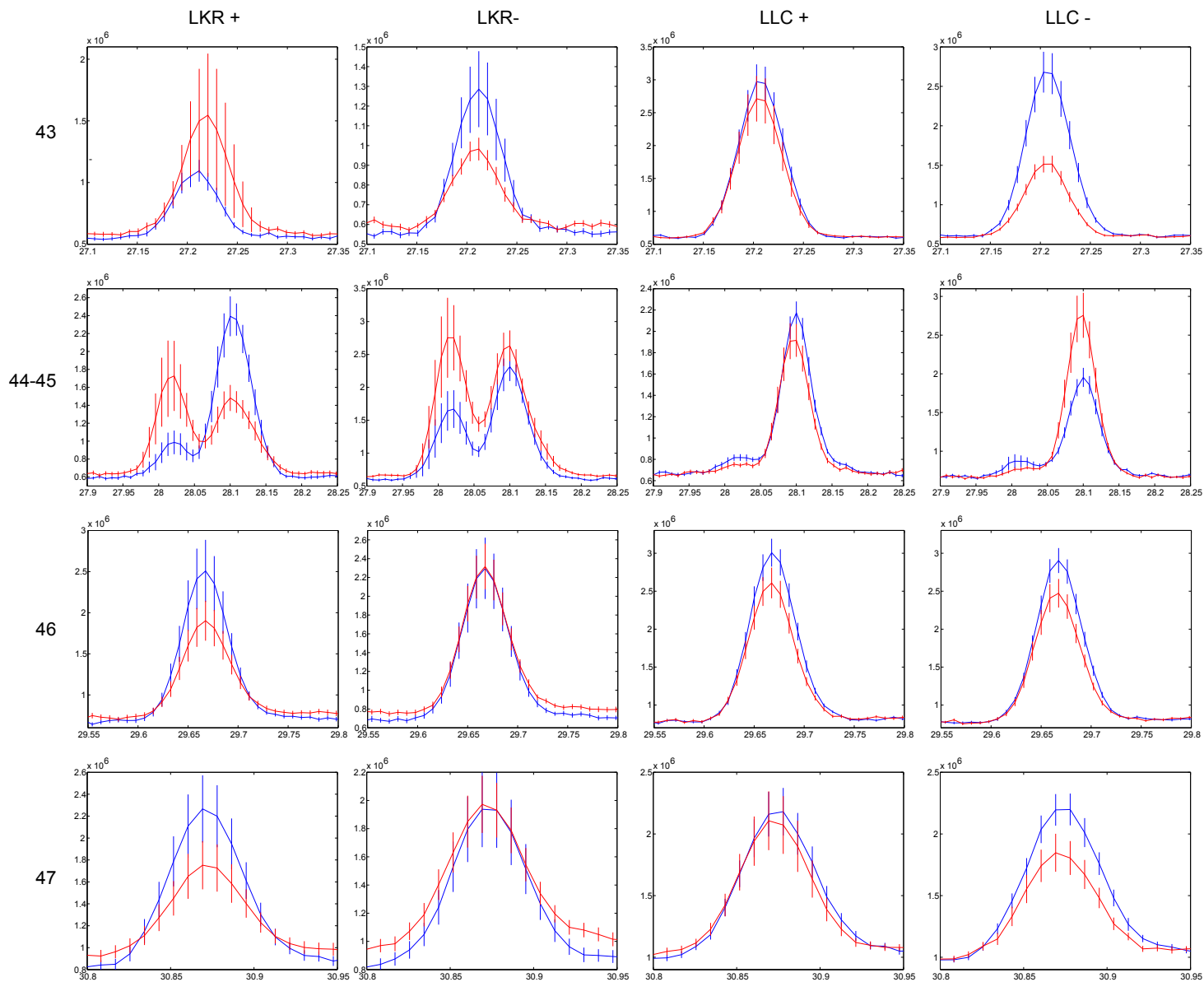

Supplement: Figure S2 — Comparison between early stage and late stage of peaks. Forty-seven peaks were selected from the TICs for further analysis. Vertical axis indicates intensity (amount) of TIC; vertical lines around mean indicate SEM at each sampling point. Blue represents the early stage whereas red represents the late stage. Horizontal axis indicates retention time. (3.99 MB PDF) [file pone.0008819.s002.pdf]

Figure S4

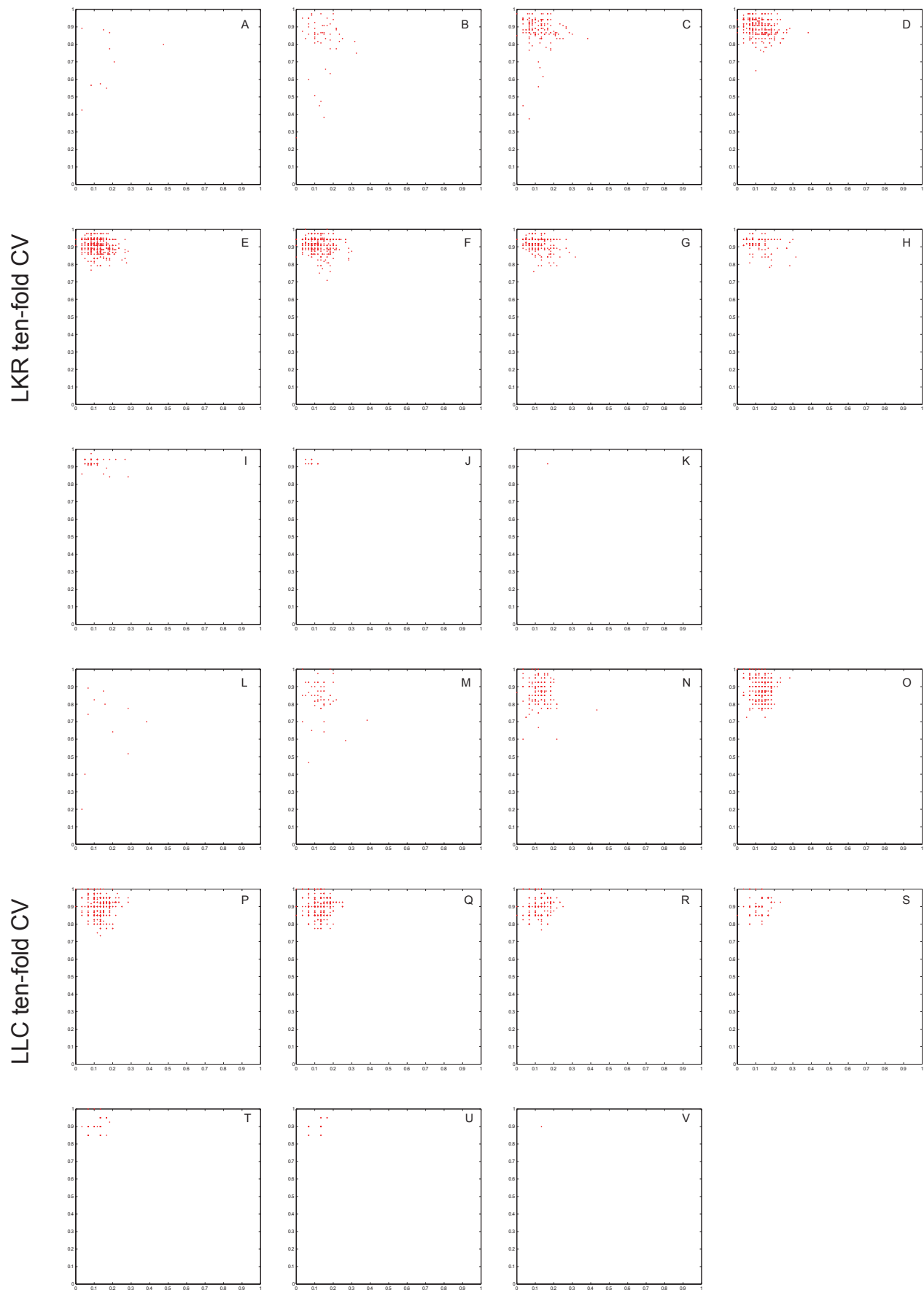

Supplement: Figure S4 — Classification performance in ROC space of paired peaks. Each box (A-K) provides an overall visual representation of ROC obtained from the ten-fold cross validation of LKR. Red dots indicate mean sensitivity (vertical axis) and mean specify (horizontal axis, 1-specificity) for each classifier. The number of paired peaks are: A, 1; B, 2; C, 3; D, 4; E, 5; F, 6; G, 7; H, 8; I, 9; J, 10; K, 11. Each box (L–V) provides an overall visual representation of ROC obtained from the ten-fold cross validation of LLC. Red dots indicate mean sensitivity (vertical axis) and mean specify (horizontal axis, 1-specificity) for each classifier. The number of paired peaks are: L, 1; M, 2; N, 3; O, 4; P, 5; Q, 6; R, 7; S, 8; T, 9; U, 10; V, 11. (0.64 MB PDF) [file pone.0008819.s004.pdf]

Figure S5

training: LKR, test: LKR

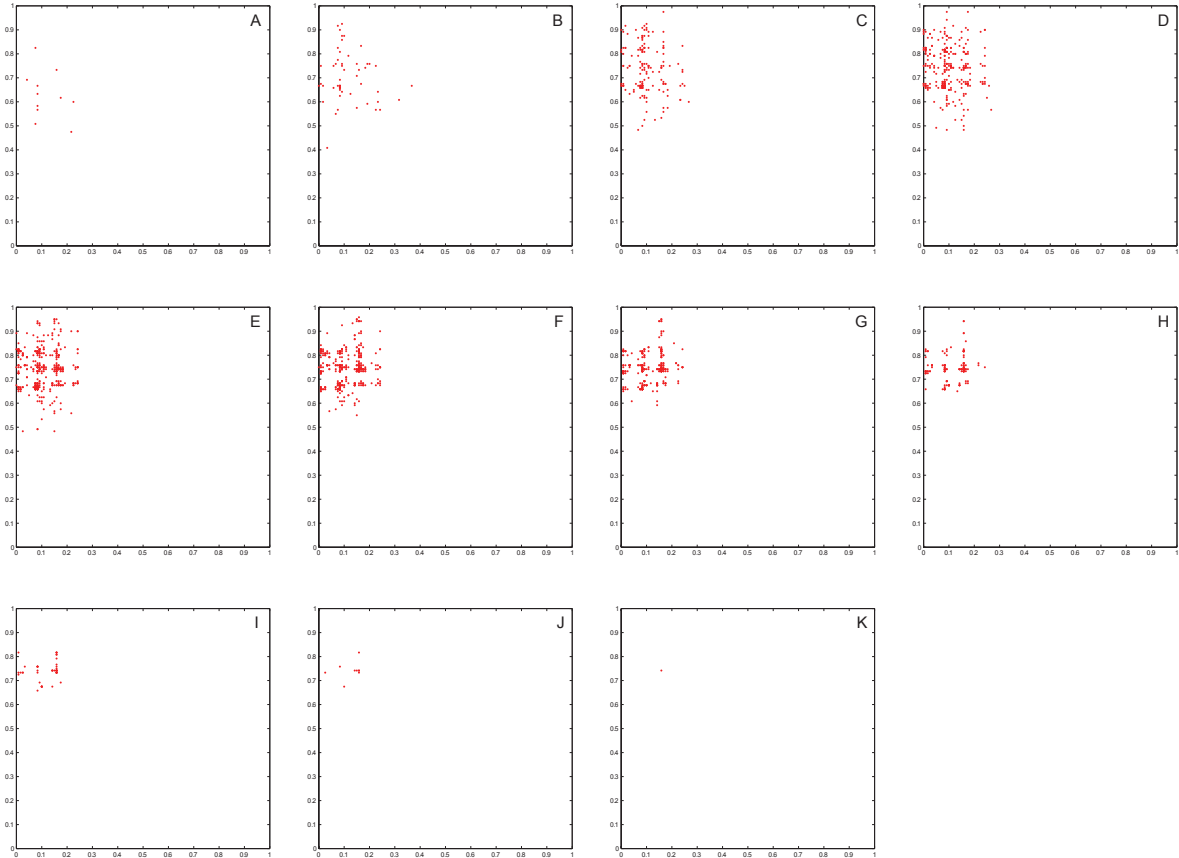

training: LLC, test: LLC

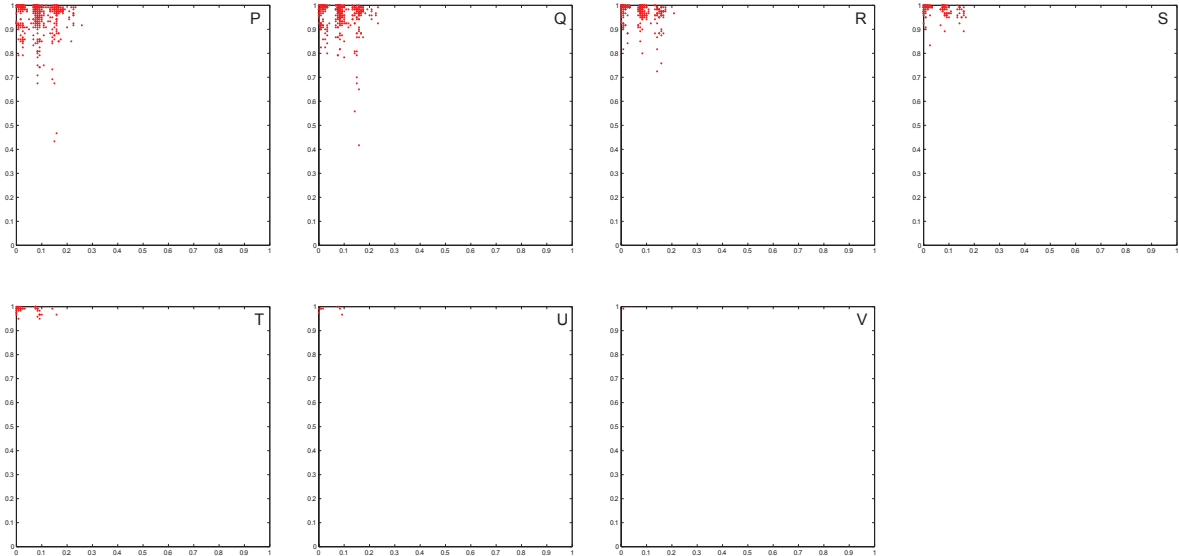

Supplement: Figure S5 — Generalization performance in ROC space of paired peaks. Each box (A–K) provides an overall visual representation of ROC obtained from generalization to the LKR test group by the LKR training group. Red dots indicate mean sensitivity (vertical axis) and mean specify (horizontal axis, 1-specificity) for each classifier. The number of paired peaks are: A, 1; B, 2; C, 3; D, 4; E, 5; F, 6; G, 7; H, 8; I, 9; J, 10; K, 11. Each box (L–V) provides an overall visual representation of ROC obtained from generalization to the LLC test group by the LLC training group. Red dots indicate mean sensitivity (vertical axis) and mean specify (horizontal axis, 1-specificity) for each classifier. The number of paired peaks are: L, 1; M, 2; N, 3; O, 4; P, 5; Q, 6; R, 7; S, 8; T, 9; U, 10; V, 11. (0.67 MB PDF) [file pone.0008819.s005.pdf]

Figure S6

training: LKR, test: LLC

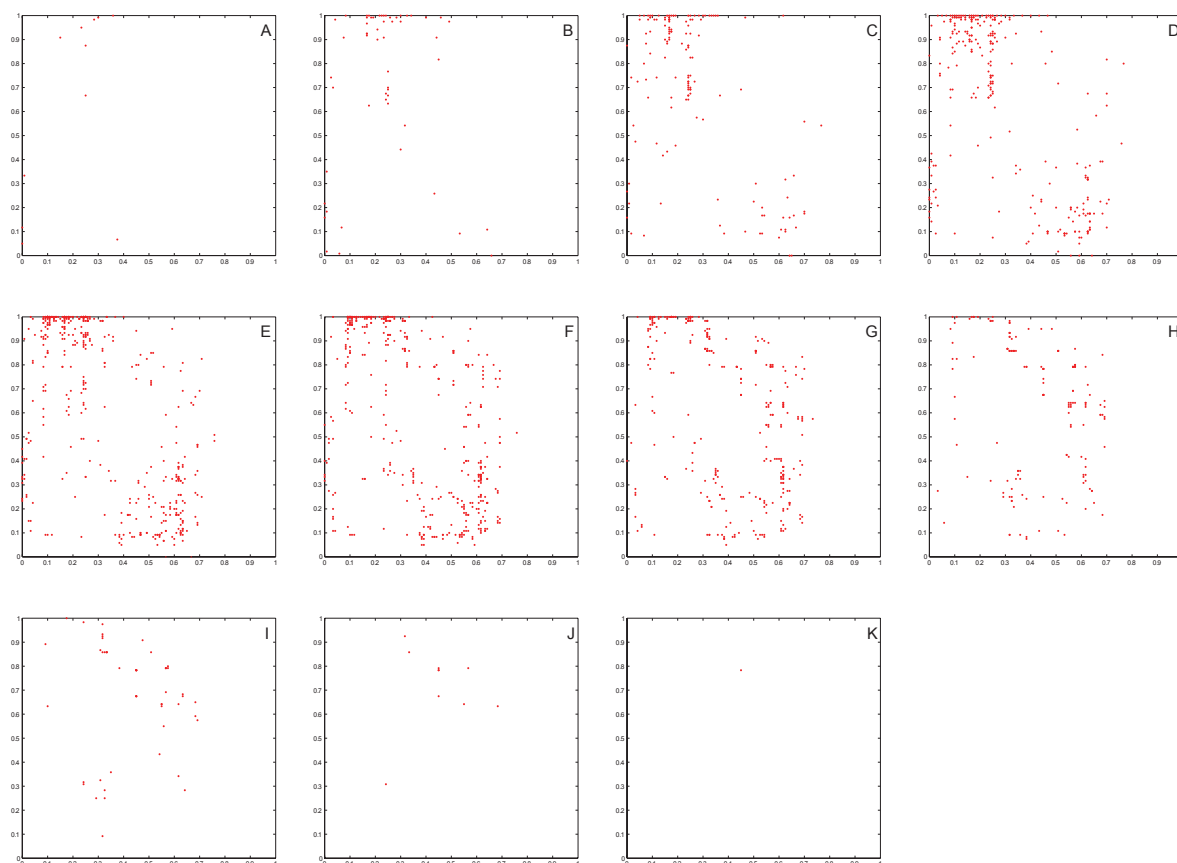

training: LLC, test: LKR

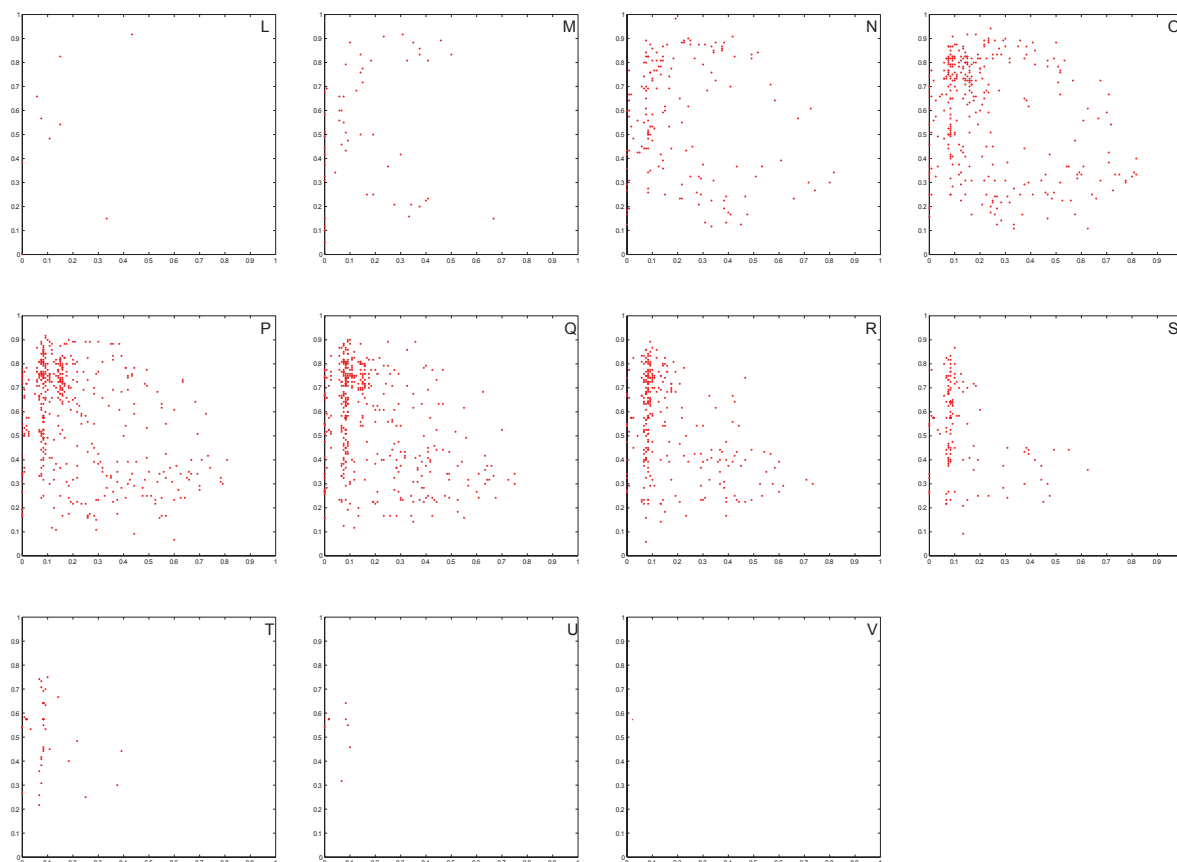

Supplement: Figure S6 — Generalization performance in ROC space of paired peaks. Each box (A–K) provides an overall visual representation of ROC obtained from generalization to the LLC test group by the LKR training group. Red dots indicate mean sensitivity (vertical axis) and mean specify (horizontal axis, 1-specificity) for each classifier. The number of paired peaks are: A, 1; B, 2; C, 3; D, 4; E, 5; F, 6; G, 7; H, 8; I, 9; J, 10; K, 11. Each box (L–V) provides an overall visual representation of ROC obtained from generalization to the LKR test group by the LLC training group. Red dots indicate mean sensitivity (vertical axis) and mean specify (horizontal axis, 1-specificity) for each classifier. The number of paired peaks are: L, 1; M, 2; N, 3; O, 4; P, 5; Q, 6; R, 7; S, 8; T, 9; U, 10; V, 11. (0.70 MB PDF) [file pone.0008819.s006.pdf]

Figure S7

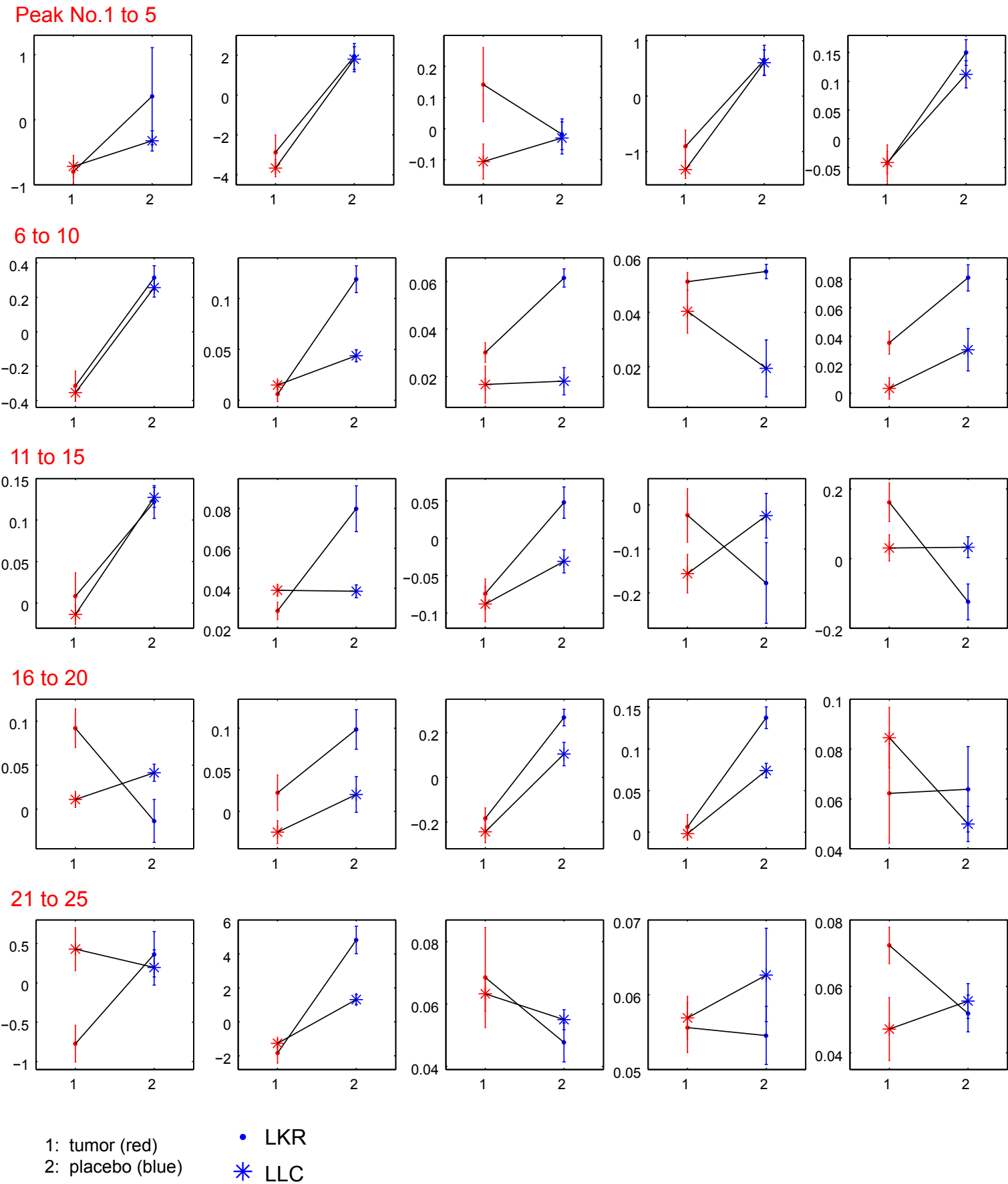

Figure S7

Peak No.26 to 30

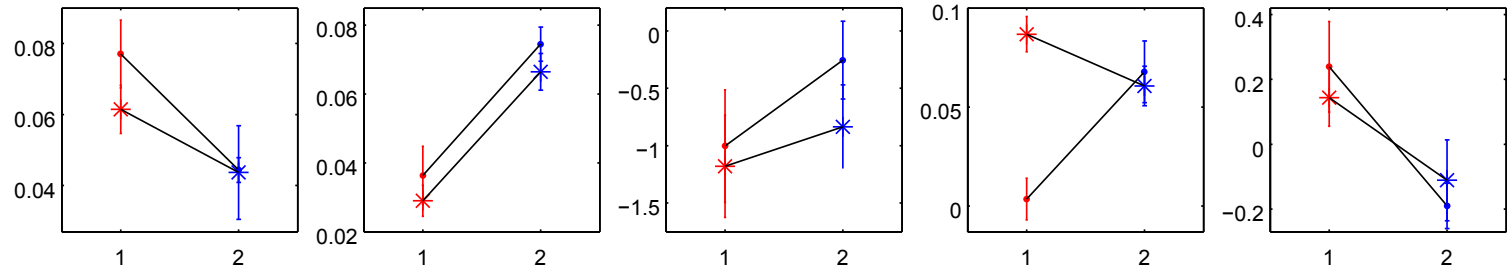

31 to 35

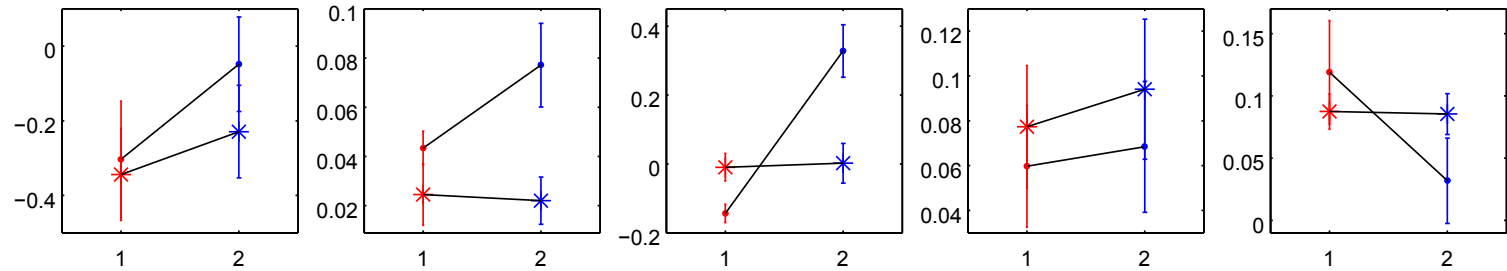

36 to 40

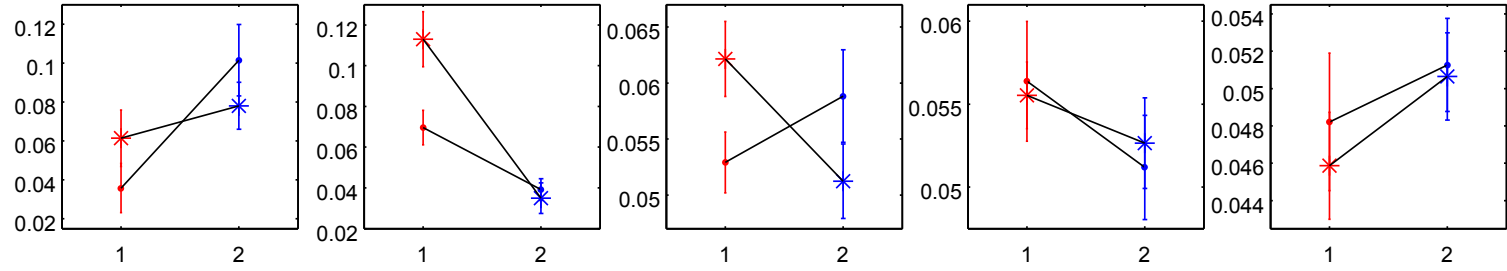

41 to 45

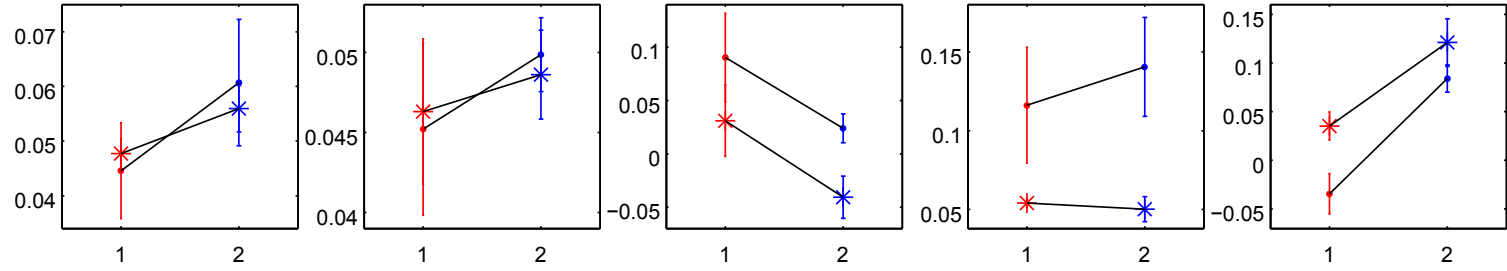

46 to 47

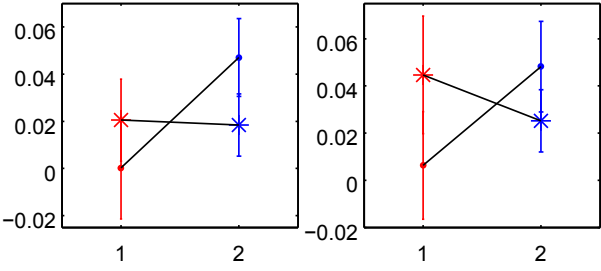

1: tumor (red)  
2: placebo (blue)

• LKR  
\* LLC

Supplement: Figure S7 — Regulatory factor determined by two-way ANOVA. Normalized intensity of subtracted peaks in a two-way ANOVA. Red: tumor, Blue: placebo, Circle: LKR, Star: LLC. (0.41 MB PDF) [file pone.0008819.s007.pdf]
