# Supplementary material for: Urinary Volatile Compounds as Biomarkers for Lung Cancer: A Proof of Principle Study Using Odor Signatures in Mouse Models of Lung Cancer
Source: PLoS One. 2010 Jan 27;5(1):e8819. doi: 10.1371/journal.pone.0008819 (PMC2811722; doi:10.1371/journal.pone.0008819)

Figure S3

Bar plot of Raw data (value=height of Late stage - height ofearly stage)_47peaks

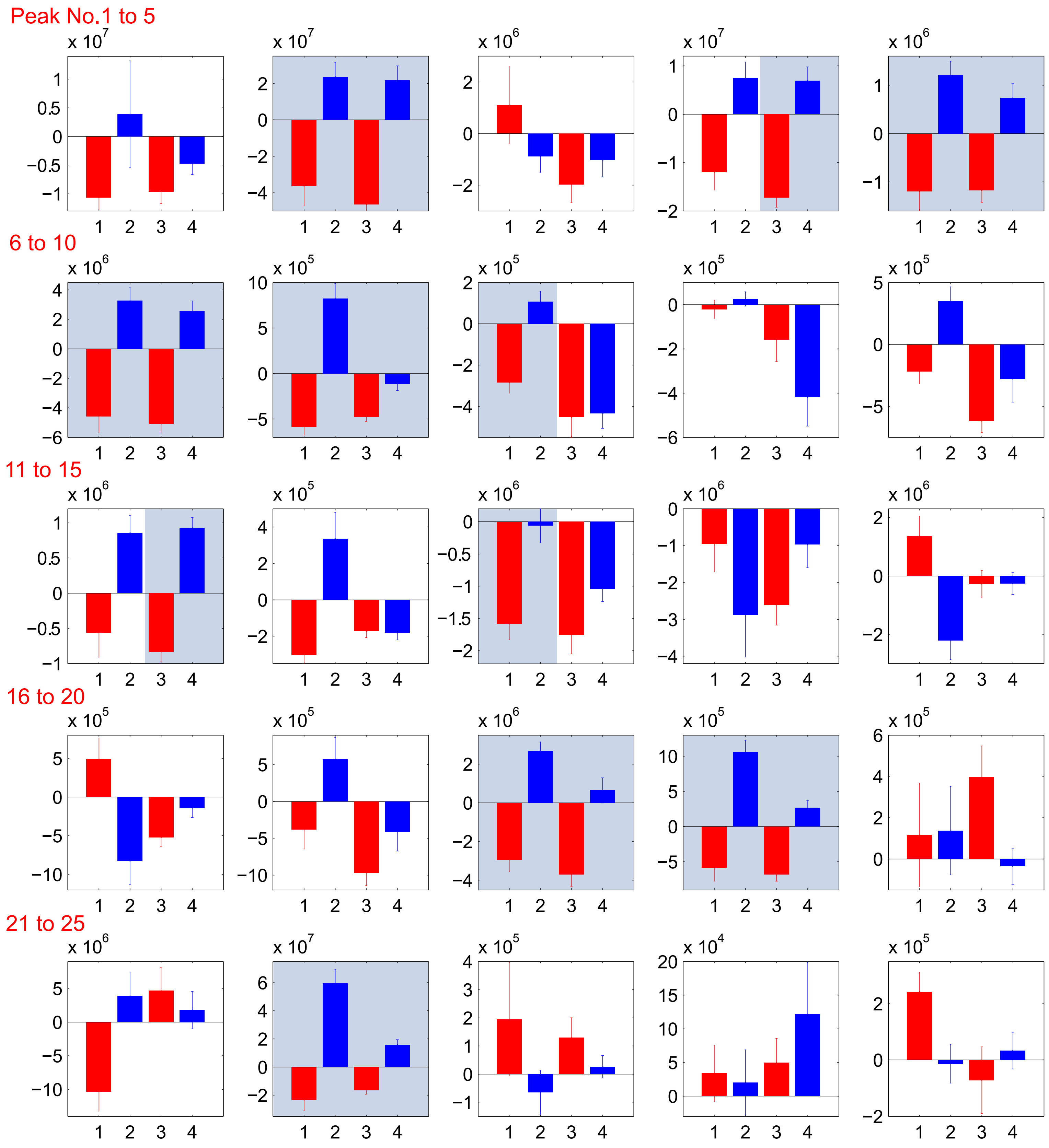

1: LKR+, 2: LKR-, 3:LLC+, 4: LLC-

Figure S3

Bar plot of Raw data (value=height of Late stage - height ofearly stage)_47peaks

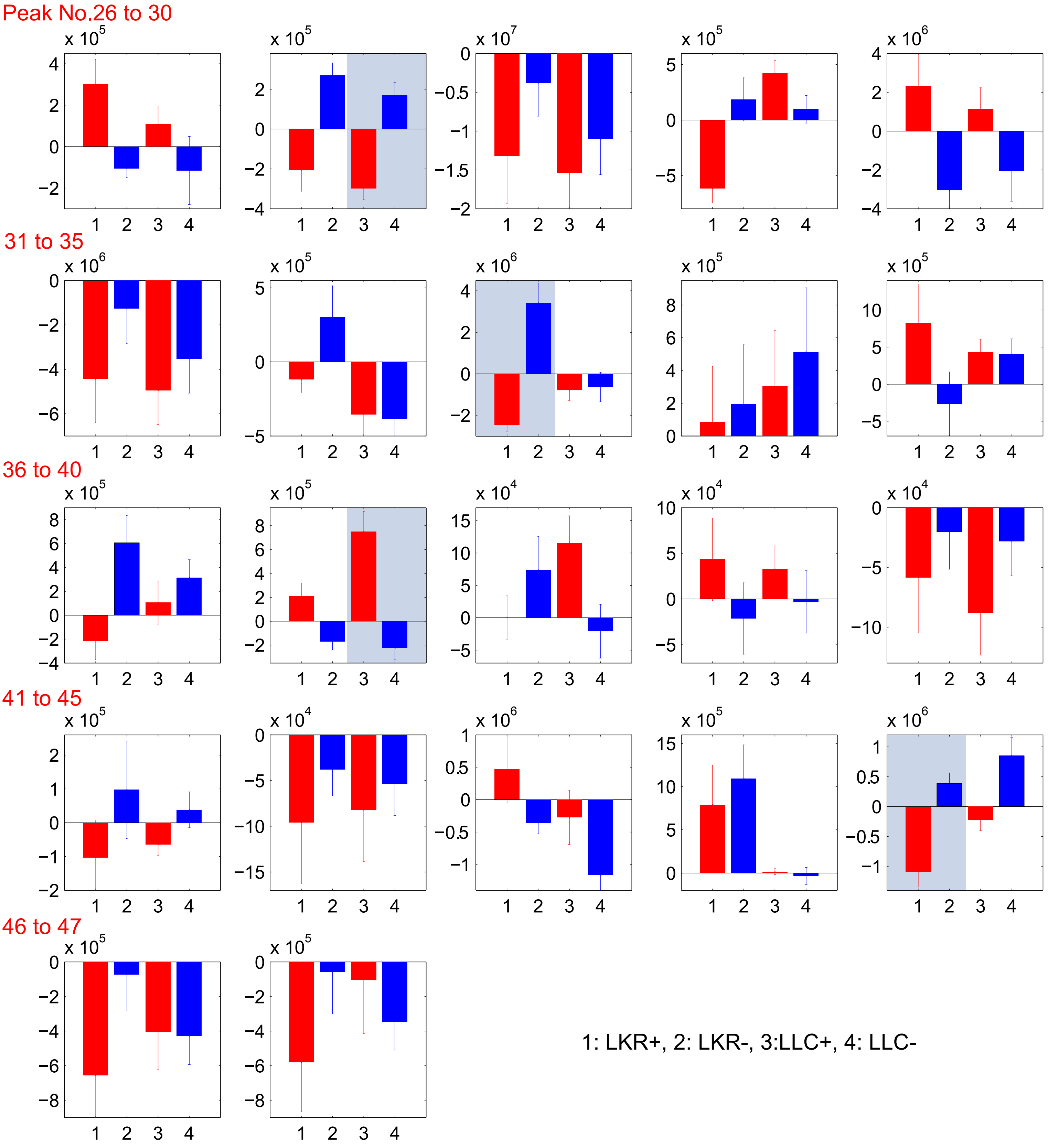

Supplement: Figure S3 — Bar plot of the intensity of the 47 peaks. Mean peak intensity is plotted for each peak. Red bars represent tumor groups whereas blue bars represent control groups. A pale blue background indicates a significant difference at P<0.0001 between tumor and control groups. It is possible to create a "bar code" representing each individual mouse. (0.47 MB PDF) [file pone.0008819.s003.pdf]
