# Supplementary material for: Urinary Volatile Compounds as Biomarkers for Lung Cancer: A Proof of Principle Study Using Odor Signatures in Mouse Models of Lung Cancer
Source: PLoS One. 2010 Jan 27;5(1):e8819. doi: 10.1371/journal.pone.0008819 (PMC2811722; doi:10.1371/journal.pone.0008819)
Supplement: Table S1 — (0.32 MB PDF) [file pone.0008819.s008.pdf]

Table S1. selected 47 peaks, compounds, and p-values

No.	RT	Compounds	LKR+vsLKR-	LLC+vsLLC-
1)	1.29	trimethylamine	0.1455	0.0353
2)	2.8	5,5-dimethyl-2-ethyltetrahydrofuran-2-ol	0.0001	0.0001
3)	4.15	chloroform	0.1896	0.3047
4)	5.1	5,5-dimethyl-2-ethyltetrahydrofuran-2-ol	0.0002	0.0001
5)	5.29	5,5-dimethyl-2-ethyltetrahydrofuran-2-ol	0.0001	0.0001
6)	6.95	5,5-dimethyl-2-ethyltetrahydrofuran-2-ol	0.0001	0.0001
7)	7.31	nitromethane	0.0001	0.0001
8)	7.99	2-heptanone	0.0001	0.886
9)	8.69	6-methyl-3-heptanone (T)	0.3654	0.0891
10)	8.83	6-methyl-3-heptanone (T)	0.0002	0.1309
11)	9.29	unkown 2 compounds	0.001	0.0001
12)	9.58	5-hepten-2-one (E or Z)	0.0003	0.8752
13)	9.77	5-hepten-2-one (E or Z)	0.0001	0.0415
14)	10.72	acetate	0.1094	0.0056
15)	10.95	*exo*-brevicomine	0.0005	0.9575
16)	11.42	acetate	0.0002	0.0139
17)	11.64	4-one-1-heptan-5-one (?)	0.0069	0.0183
18)	11.88	2-acetyl-1-pyrroline	0.0001	0.0001
19)	12.94	2-isopropyl-4,5-dihydrothiazole	0.0001	0.0001
20)	13.38	butoxyethanol (from paint)	0.9477	0.0068
21)	13.52	3,4-dehydro-*exo*-brevicomin	0.0028	0.51
22)	14.79	2-*sec*-butyl-4,5-dihydrothiazole	0.0001	0.0001
23)	16.05	unknown exogeneous compounds and silicon	0.2277	0.2072
24)	16.32	benzaldehyde	0.8652	0.4209
25)	16.48	linalool	0.0019	0.4585
26)	17.09	isobutylic acid	0.004	0.2501
27)	17.2-18.1	6-hydroxy-6-methyl-3-heptanone	0.0005	0.0001
28)	18.75	*trans*-*β*-farnesene	0.1922	0.5314
29)	18.87	acetophenone	0.0007	0.0523
30)	19.06	isovaleric and 2-methylbutyric acids	0.0176	0.1137
31)	20.39	*α*-farnesene	0.1902	0.469
32)	20.59	methoxy-phenyl-oxime	0.0965	0.8416
33)	21.79	*o*-toluidine	0.0001	0.8743
34)	22.82	unknown (paint or wall)	0.81	0.6843
35)	22.89	unknown (paint or wall)	0.113	0.9062
36)	23.66-23.73	dimethyl sulfone	0.0023	0.356
37)	24.18	2-ethyl hexanoic acid	0.0018	0.0001
38)	24.26	unknown (SPME fiber related)	0.1966	0.0676
39)	24.37	dodecanal	0.278	0.3169
40)	26.51	alkyl acetyl pyrolle	0.4225	0.1432
41)	26.61	*p*-cresol	0.2285	0.0711
42)	26.93	unknown	0.3432	0.6114
43)	27.28	cedrol or epicedrol	0.1462	0.0405
44)	28.09	*p*-ethyl phenol	0.6322	0.6148
45)	28.16	*N*-phenyl formamide	0.0001	0.0023
46)	29.75	acetate	0.0404	0.8936
47)	30.95	acetate or alcohol	0.1488	0.4033

P<0.0001	
P<0.001	
P<0.01	
P<0.1	
P<1	
