# Supplementary material for: Urinary Volatile Compounds as Biomarkers for Lung Cancer: A Proof of Principle Study Using Odor Signatures in Mouse Models of Lung Cancer
Source: PLoS One. 2010 Jan 27;5(1):e8819. doi: 10.1371/journal.pone.0008819 (PMC2811722; doi:10.1371/journal.pone.0008819)
Supplement: Table S2 — (0.31 MB PDF) [file pone.0008819.s009.pdf]

Table S2. Summary of highest score (full)

LKR ten-fold CV	Accuracy		Sensitivity		Specificity	
Peak No. 7, 13, 22	0.98	0.02	0.975	0.025	1	1
Peak No. 8, 13, 18, 19, 22 , 45	0.98	0.02	1	0	0.95	0.05

LLC ten-fold CV	Accuracy		Sensitivity		Specificity	
Peak No. 5, 11, 19, 37	1	0	1	0	1	0
Peak No. 2, 4, 6, 19, 37	1	0	1	0	1	0
Peak No. 2, 4, 6, 7, 19, 37	1	0	1	0	1	0

LKR to LKR	Accuracy		Sensitivity		Specificity	
Peak No. 7, 8, 13	0.95	0.0056	0.9167	0	0.9833	0.0111
Peak No. 13, 33, 45	0.95	0.0083	0.9	0.0167	1	0
Peak No. 8, 13, 33, 45	0.95	0.0083	0.9	0.0167	1	0

LKR to LLC	Accuracy		Sensitivity		Specificity	
Peak No. 13, 22, 33, 45	0.9833	0.0167	1	0	0.9667	0.0333
Peak No. 7, 13, 22, 33, 45	0.9833	0.0167	1	0	0.9667	0.0333
Peak No. 8, 13, 22, 33, 45	0.9833	0.0167	1	0	0.9667	0.0333
Peak No. 13, 19, 22, 33, 45	0.9833	0.0167	1	0	0.9667	0.0333
Peak No. 7, 8, 13, 22, 33, 45	0.9833	0.0167	1	0	0.9667	0.0333
Peak No. 7, 13, 19, 22, 33, 45	0.9833	0.0167	1	0	0.9667	0.0333
Peak No. 8, 13, 19, 22, 33, 45	0.9833	0.0167	1	0	0.9667	0.0333

LLC to LLC	Accuracy		Sensitivity		Specificity	
	1	0	1	0	1	0
2, 6, 19,37,	6, 7, 22,27,37,		2, 4, 5, 6, 7, 22,37,			
4, 5, 19,37,	6, 11,19,22,37,		2, 4, 6, 7, 19,22,37,			
5, 22,27,37,	2, 4, 6, 7, 19,37,		2, 4, 6, 7, 19,27,37,			
6, 7, 22,37,	2, 4, 6, 7, 22,37,		2, 4, 6, 7, 22,27,37,			
6, 22,27,37,	2, 4, 6, 19,22,37,		2, 4, 6, 19,22,27,37,			
2, 4, 6, 19,37,	2, 4, 6, 19,27,37,		4, 5, 7, 11,19,22,37,			
2, 4, 6, 22,37,	2, 4, 6, 22,27,37,		4, 5, 7, 11,22,27,37,			
2, 6, 7, 19,37,	2, 6, 7, 19,22,37,		4, 5, 11,19,22,27,37,			
2, 6, 19,22,37,	4, 5, 7, 11,22,37,		5, 7, 11,19,22,27,37,			
4, 5, 11,19,37,	4, 5, 7, 19,27,37,		2, 4, 5, 6, 7, 19,22,37,			
4, 5, 19,22,37,	4, 5, 11,19,22,37,		2, 4, 5, 6, 7, 22,27,37,			
4, 5, 19,27,37,	4, 5, 11,19,27,37,		2, 4, 6, 7, 19,22,27,37,			
4, 5, 22,27,37,	5, 7, 11,19,22,37,		4, 5, 7, 11,19,22,27,37,			
5, 7, 11,19,37,	5, 11,19,22,27,37,		2, 4, 5, 6, 7, 19,22,27,37,			
5, 11,19,22,37,	6, 11,19,22,27,37,					

LLC to LKR	Accuracy		Sensitivity		Specificity	
Peak No. 5, 11, 22, 27, 37	0.9125	0.0042	0.9167	0	0.9083	0.0083
