# Supplementary material for: Urinary Volatile Compounds as Biomarkers for Lung Cancer: A Proof of Principle Study Using Odor Signatures in Mouse Models of Lung Cancer
Source: PLoS One. 2010 Jan 27;5(1):e8819. doi: 10.1371/journal.pone.0008819 (PMC2811722; doi:10.1371/journal.pone.0008819)
Supplement: Table S3 — (0.24 MB PDF) [file pone.0008819.s010.pdf]

Table S3. results of two-way ANOVA

Peak No.	tumor	cell line	interaction
1	0.006	0.0002	0.0001
2	0	0.0109	0.0751
3	0.5924	0.651	0.5129
4	0	0.0182	0.3372
5	0	0.089	0.0845
6	0	0.006	0.0461
7	0	0.0004	0.0006
8	0.0037	0.0039	0.2488
9	0.2298	0.0771	0.8312
10	0.0013	0.0773	0.8142
11	0	0.0035	0.0538
12	4.34E-07	3.43E-05	9.00E-07
13	0.0001	0.2905	0.525
14	0.8392	0.0039	0.1758
15	0.0017	0.1097	0.2297
16	0.0165	0.0028	0.0523
17	0.002	0.0058	0.5868
18	0	0.0101	0.0511
19	0	0.2312	0.2286
20	0.3289	0.8523	0.9754
21	0.0614	0.075	0.1004
22	0	0.3948	0.0025
23	0.1075	0.3441	0.3573
24	0.5889	0.8017	0.2713
25	0.3587	0.1628	0.4162
26	0.0062	0.4777	0.356
27	0	0.0524	0.0042
28	0.1797	0.2947	0.4498
29	0.0387	0.0002	0.0004
30	0.0012	0.3098	0.0896
31	0.1654	0.3253	0.5707
32	0.097	0.0005	0.0028
33	0	0.0429	0.0018
34	0.6692	0.4007	0.8704
35	0.126	0.6038	0.275
36	0.0074	0.3079	0.7524
37	0	0.0592	0.2006
38	0.4488	0.2682	0.1949
39	0.1295	0.0531	0.2808
40	0.1705	0.1104	0.8832
41	0.0531	0.0022	0.0904
42	0.3634	0.1536	0.6547
43	0.0175	0.1218	0.5151
44	0.659	0.1117	0.1199
45	0	0.0519	0.364
46	0.2142	0.1582	0.9846
47	0.5943	0.3525	0.7836
